# Accountability Assessment of Source-Specific Impacts of Regulations on Emissions and Air Quality Using Positive Matrix Factorization

**DOI:** 10.1021/acs.est.4c12511

**Published:** 2025-04-24

**Authors:** Ziqi Gao, Eric J. Mei, Xin He, Stefanie Ebelt, David Q. Rich, Armistead G. Russell

**Affiliations:** 1 School of Civil and Environmental Engineering, Georgia Institute of Technology, Atlanta, Georgia 30332, United States; 2 Department of Public Health Sciences, University of Rochester School of Medicine and Dentistry, Rochester, New York 14642, United States; 3 Gangarosa Department of Environmental Health, Rollins School of Public Health, Emory University, Atlanta, Georgia 30322, United States

**Keywords:** accountability, source apportionment, EGUs, mobile sources, GAM

## Abstract

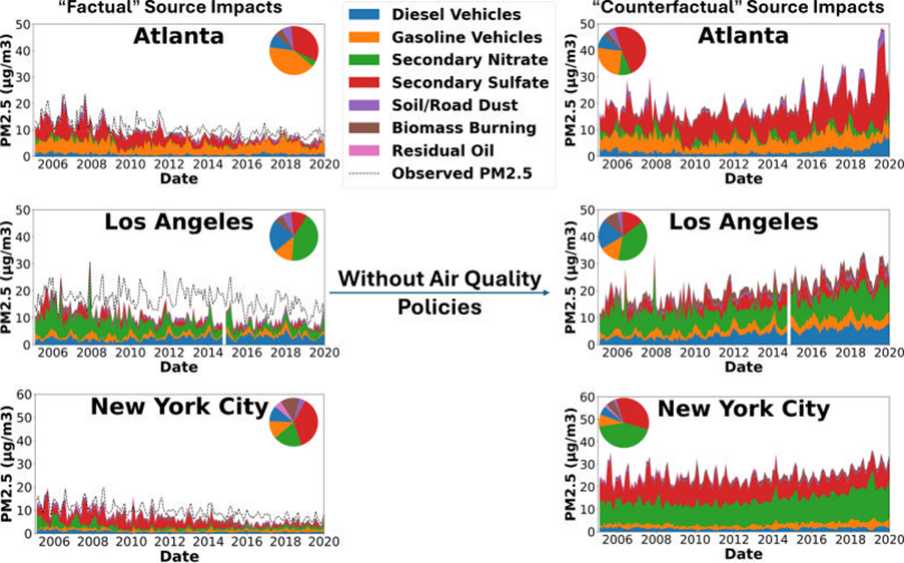

Emission controls targeting electric generating units (EGUs) and mobile sources have been implemented for decades to mitigate PM_2.5_ concentrations. Impacts of emission controls on source-apportioned PM_2.5_ concentrations (diesel/gasoline vehicles, biomass burning, secondary nitrate, secondary sulfate, soil/road dust, and residual oil estimated via positive matrix factorization) across three U.S. highly urbanized regions—Atlanta, New York City, and the South Coast Air Basin (SoCAB)—from 2005 to 2019 were evaluated. We considered major controls on EGUs, mobile sources, ports, and heating fuel. Daily counterfactual source-apportioned PM_2.5_ concentrations without emission controls were estimated based on meteorological indicators and counterfactual emissions using the generalized additive model. Results indicate that emission controls reduced the PM_2.5_ concentrations by 65–85% across all regions. Secondary sulfate concentrations without EGU controls would be 4.8 times higher, and diesel-vehicle-related PM_2.5_ would increase 6.8 times without mobile controls in Atlanta. Secondary inorganic aerosols in New York City would increase 5-fold from 1.92 to 10.5 μg/m^3^, shifting the dominant PM_2.5_ contributors. Seasonal trends in the counterfactual PM_2.5_ concentrations were similar to the actual trends, but the peaks in the counterfactual scenario were clearer than those with emission controls.

## Abstract 2

Emission regulations targeting EGUs and mobile sources emissions have effectively reduced PM_2.5_ concentrations in major U.S. cities. This study quantifies how these regulations affected source-apportioned PM_2.5_ over time.

## 1 Introduction

Fine particulate matter (PM_2.5_) has a variety of adverse human health, economic, and environmental impacts.^1^ Its formation is a complex process involving direct emissions and secondary creation through atmospheric chemical reactions, and it consists of various chemical components.^2^ The sources of PM_2.5_ vary by location and season, and dominant sources and the contributions of each source to PM_2.5_ concentrations have changed due to emission regulations.^3−7^ Major US emission regulations on sources such as electrical generating units [EGUs; i.e., power plants; regulations include the Acid Rain Program (ARP), NOx State Implementation Plan (NOx SIP Call), NOx Budget Trading Program (NBP), Clean Air Interstate Rule (CAIR) and Cross-State Air Pollution Rules (CSAPR)] have reduced nitrogen oxide (NOx) and sulfur dioxide (SO_2_) emissions, precursors to secondary nitrate and sulfate aerosols, by 88 and 95%, respectively, according to the US Environmental Protection Agency (EPA) Clean Air Markets Program Data (CAMPD) progress summary.^8^ Major gasoline vehicle and diesel engine emission regulations (including Tier 2 and 3 vehicle emissions and gasoline sulfur programs^9,10^ and the Heavy-Duty Diesel Program^11^) have reduced CO, NOx, SO_2_, volatile organic compound (VOC), and primary PM_2.5_ emissions and affected ammonia (NH_3_) emissions via catalytic converters, diesel exhaust fluid injection, diesel particulate filters, and fuel standards (e.g., lower sulfur levels), which are precursors to various PM_2.5_ chemical constituents and ozone. Previous studies showed that ozone, PM_2.5_, and PM_2.5_ chemical species concentrations would have increased without these EGU and mobile regulations.^7^ These studies highlight the importance of understanding the contribution of regulations to reducing PM_2.5_ concentrations that can be attributed to individual sources.

Receptor models such as positive matrix factorization (PMF) and chemical mass balance models quantitatively estimate contributions from different pollution sources by linking ambient measurements to source emissions compositions. Our study used PMF-derived source impacts and generalized additive models (GAMs) to analyze the relationship among source-apportioned PM_2.5_ concentrations, emissions, and meteorological factors. We use these relationships to quantify the emission controls’ impacts on dominant sources and their relative contributions to PM_2.5_ concentrations from 2005 to 2019 in Atlanta, New York City, and the South Coast Air Basin (SoCAB). These urbanized and highly polluted cities are in different U.S. regions, each with unique meteorological conditions and unique source characteristics.

As part of an accountability assessment of the impact of regulations on air quality and health, GAMs were used to compute daily counterfactual source-apportioned PM_2.5_ concentrations (including PM_2.5_ from gasoline, diesel, residual oil, soil and road dust, biomass burning, secondary nitrate, and secondary sulfate sources) without the studied emission controls. We then compared the counterfactual concentrations to the actual source-apportioned PM_2.5_ impacts estimated using PMF. The differences between the actual and counterfactual concentrations show the impact of emission control policies on PM_2.5_ concentrations from different sources. The factual–counterfactual approach extends beyond a more traditional trend analysis to explicitly allow for comparing the impact of specific regulations.

## 2 Data and Methods

We developed GAMs for the actual PM_2.5_ concentrations from each source factor using meteorology and actual emissions. We then estimated the emissions from power plants, mobile sources, and residual oil without control policies (i.e., the counterfactual case). Using the estimated counterfactual emissions and observed meteorology, we predicted the counterfactual PM_2.5_ concentrations to assess the impact of emission controls on each source factor of PM_2.5_ concentrations.

### 2.1 Data

#### 2.1.1 Observation Data Sets

Meteorological data, including temperature, relative humidity, wind speed, dew point temperature, precipitation, and sea level pressure (SLP), were collected for Atlanta (South DeKalb site), New York City (Queens, Manhattan, and Bronx sites), and SoCAB (Los Angeles North Main Street [LA Main Street] and Rubidoux sites). These six sites were selected because they are the only CSN sites providing long-term PM_2.5_, PM_2.5_ species, and gaseous species data covering the full study period from 2005 to 2019 in each of the cities (although there are gaps in some of the data). These data were obtained from the National Centers for Environmental Information (NCEI) and the National Oceanic and Atmospheric Administration (NOAA) archives, as well as the California Air Resources Board (CARB) archives.^12,13^ Primary pollutant emissions on an annual basis for each emission source were obtained from CARB and the EPA’s Air Pollutant Emissions Trends Data, which projects from 2017 emissions (https://www.epa.gov/air-emissions-inventories/air-pollutant-emissions-trends-data, last access: 3/22/2022).^14,15^ Daily emissions of NOx and SO_2_ from EGUs in Georgia, New York, New Jersey, Connecticut, and several upwind states contributing to elevated EGU-related pollutants in New York (i.e., Ohio, West Virginia, Pennsylvania, Illinois, Indiana, Kentucky, and Michigan) were retrieved from the EPA’s Clean Air Markets Program Data (CAMPD).^8^ Estimated mobile emissions were modeled using the Motor Vehicle Emission Simulator (MOVES3) and CARB’s Emission FACtors (EMFAC).

**Figure 1:**
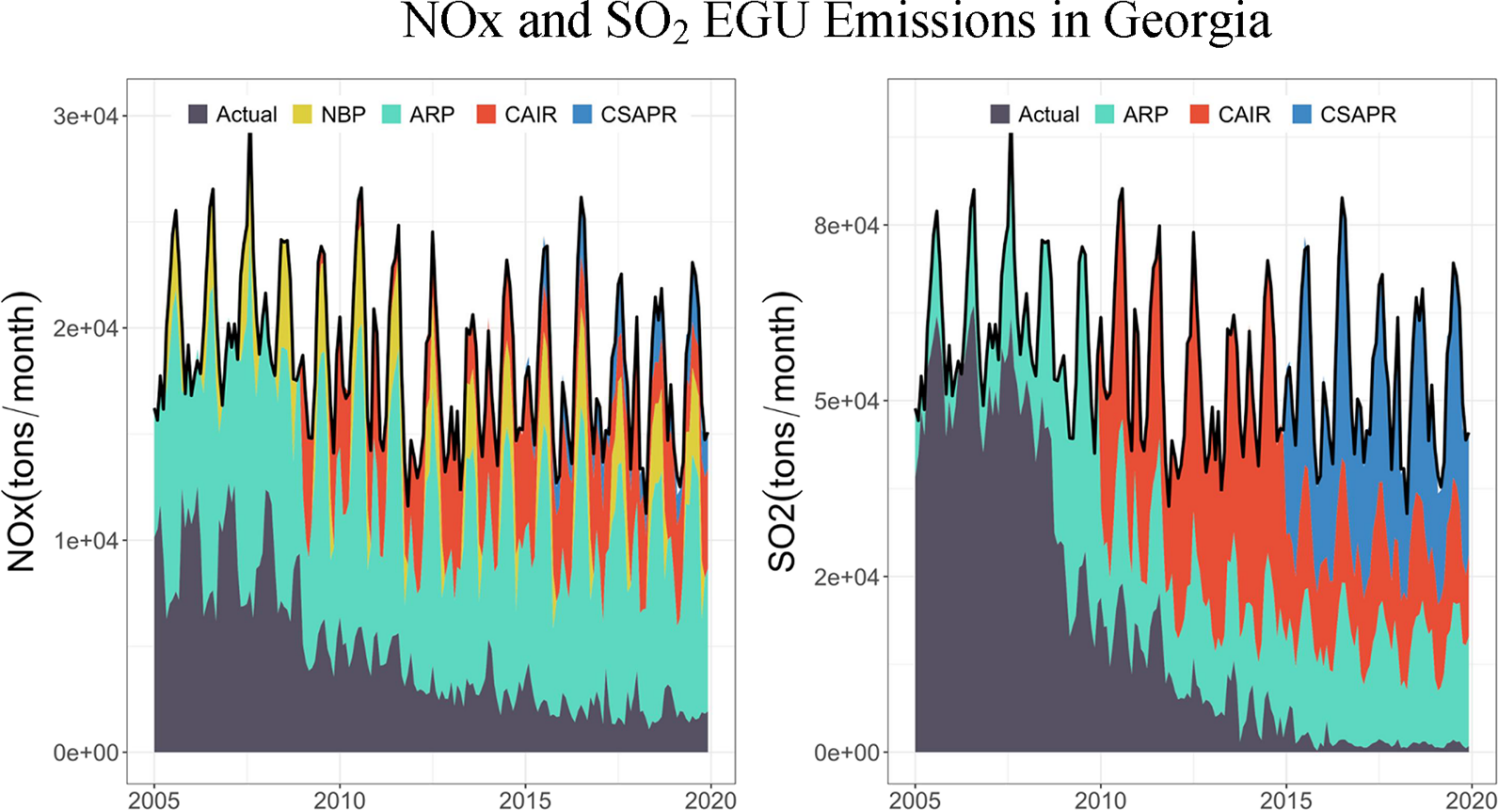
Change in NOx and SO_2_ emissions from EGU sources in Georgia. The gray area shows the reported (actual) emissions, the yellow area is the modeled impact of the NOx SIP call and NBP, the green is ARP, the red is CAIR, the blue is CSAPR, and the black line shows the counterfactual EGU emissions. Adapted from ref (17). Available under CC-BY 4.0. Copyright 2024 Elsevier.

The limit on NOx and SO_2_ EGU emissions due to regulations, including the Acid Rain Program (ARP), NOx State Implementation Plan (NOx SIP Call), NOx Budget Trading Program (NBP), Clean Air Interstate Rule (CAIR), and Cross-State Air Pollution Rules (CSAPR), varied seasonally in both Georgia (GA) and New York (NY).^17^ The cumulative impacts of these regulations resulted in a significant reduction of NOx and SO_2_ emissions from EGU sources, achieving reductions of approximately 88 and 97% in GA and about 96 and 99% in NY, respectively (Figure 1 and Figure S1).

Estimated emissions from mobile sources without emission controls were considerably higher than actual emissions (Figure S2). In Georgia, diesel programs significantly reduced primary PM_2.5_ emissions, whereas gasoline programs decreased other species’ emissions. Diesel programs substantially reduced NOx and PM_2.5_ emissions, while gasoline controls predominantly affected NH_3_ and VOC emissions in the SoCAB. Port emission regulations considerably decreased SO_2_ emissions, followed by diesel programs (13%). Furthermore, diesel programs significantly reduced PM_2.5_ emissions in New York, while gasoline programs had greater effects on other species’ emissions than diesel controls. Gasoline controls are modeled to have led to decreases in NH_3_ emissions across all three areas. This reduction is primarily due to stricter vehicle emission standards, reformulated gasoline programs, and control technology advancements, which reduce NH_3_ formation.^15,16^ Also, the increased adoption of gasoline hybrid technologies and electric vehicles further reduces the level of NH_3_ emissions. In contrast, counterfactual NH_3_ emissions without diesel emission controls in the SoCAB were lower than the factual emissions. This is due to NH_3_ emissions from diesel selective catalytic reduction (DSCR) systems in diesel vehicles, which introduce NH_3_ as a byproduct of the NOx reduction. Without these diesel controls, NH_3_ emissions would have been lower, despite potentially higher NOx emissions.^18,19^

Regulations on stationary fuel combustion in New York City mainly targeted the emissions of PM_2.5_, NOx, and SO_2_ from large building heating systems burning No. 4 or No. 6 oil^20,21^ and the effects of the North American Emissions Control Area.^22^ However, the reductions in these species’ emissions were smaller than those from EGU and mobile sources. Between 2005 and 2019, the estimated reductions in emissions of NOx, SO_2_, and primary PM_2.5_ from stationary diesel sources, in terms of overall emissions in New York, were approximately 2, 27, and 0.15%, respectively (Figure S2)

#### 2.1.2 Source-Apportioned PM_2.5_

PMF is a receptor-oriented source apportionment model that does not require predefined source profiles.^23−25^ Details of the PMF application used in this study are available in Stanimirova et al. (2023; 2024).^26,27^ The dispersion-normalized PMF method for the PM_2.5_ source apportionment was applied at all six sites. In the SoCAB, nine factors were identified, including fresh and aged sea salt, secondary sulfate, secondary nitrate, diesel and gasoline vehicles, soil/road dust, biomass burning, and pyrolyzed organic carbon. Ten sources were identified in New York City, the additional source being residual oil, and nine sources were identified in Atlanta, where copper replaced fresh sea salt.

### 2.2 Methods

#### 2.2.1 Generalized Additive Modeling

GAM handles both linear and nonlinear relationships between air pollutant concentrations and emissions and meteorology with splines.^28−33^ Also, it can show the feature importance and contribution of each indicator to the response variable. The general GAM equation is as follows:
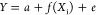
where *a* is the intercept, *e* is the error term, and *f*(*X*_i_) represents a flexible choice of functions (e.g., linear, splines, etc.).

The GAMs for each PMF factor included three different kinds of indicators: emissions from various sources, surface meteorology, and categorical indicators (e.g., day of year and day of week). The categorical indicators add daily and weekly variations to annual emissions to consider weekend/weekday impacts on source-apportioned PM_2.5_ concentrations. Previous studies discussed the advantages and limitations of GAM and compared the model performance of GAM with other traditional statistical methods and machine learning models.^34−36^ Before building the models, we did feature selection to avoid concurvity, reduce model complexity, and increase model accuracy. We applied cubic splines to emissions and meteorological indicators to show the possible nonlinear relationship between emissions/meteorology and source-apportioned PM_2.5_. All the models were built with the R “mgcv” package.^29,37−40^

Multiple statistical metrics, including the correlation coefficient (*R*^2^), mean bias (MB), and root-mean-square error (RMSE), were used to assess model performance. To further evaluate overfitting and model performance, we used a 10-fold cross-validation method, in which 90% of the data was used as training data, and the remaining 10% was used as testing data. This process was repeated 10 times.

The impacts of emission changes were calculated by taking the difference between the GAM-predicted concentrations with the counterfactual and actual estimated emissions. To calculate counterfactual concentrations, the difference between the GAM-predicted concentrations in the two scenarios is added to the observed air quality (eqs 1 and 2). This method is used because health models are typically trained on observed air pollutant concentrations rather than on air quality model predictions. This method can remove errors arising from bias in the models and ensure consistency with exposure–response relationships used in health assessments by calibrating model-predicted counterfactual air pollutant concentrations to observations.
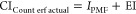
1
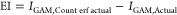
2where CI_Counterfactual_ is the calibrated counterfactual source-apportioned PM_2.5_ concentrations without emission controls, *I*_PMF_ is the observed impact found using PMF, EI is emission impacts, and *I*_GAM, Counterfactual_ and *I*_GAM, Actual_ are the impacts predicted with the GAM model for that factor using the observed meteorological data and the (estimated) counterfactual emissions without regulatory impacts on emissions and (estimated) actual emissions with the actual regulations in place.

Monte Carlo sampling was applied to estimate the uncertainty in the estimated counterfactual emissions, which is detailed in the previous study.^17^ A 95% confidence interval was used to show the uncertainty of the GAMs, which was calculated by adding and subtracting twice the standard error.

## 3 Results

### 3.1 Observed Contribution of Sources to PM_2.5_

PMF modeling led to a varying number of identified factors and their contributions to PM_2.5_ in Atlanta,^27^ the SoCAB,^26^ and New York City.^21,41^ In Atlanta, the dominant contributors were secondary sulfate (31% on average over these 15 years) and gasoline vehicles (43%) (Figure S3). Secondary sulfate contributions to PM_2.5_ decreased mainly due to regulations of EGUs (from around 43 to 12%) (Figure S3) and the impact of the 2008 recession and the simultaneous introduction of low-cost fracked natural gas.^41^ The annual average mass attributed to gasoline vehicles dropped from 4.47 to 2.69 μg/m^3^, but the fraction of PM_2.5_ that factor comprised increased from 33 to 60% due to decreases in other factor contributions (mainly secondary sulfate). Secondary sulfate, soil, and road dust concentrations were highest in the summer, while secondary nitrate concentrations were highest in the winter. Other sources did not show such seasonal trends.

The dominant source factors for PM_2.5_ in SoCAB were secondary nitrate (38% at the LA N Main St. site and 41% at the Rubidoux site) and diesel vehicles (28% at the LA Main St. site and 21% at the Rubidoux site) (Figure S3). However, the secondary nitrate factor’s concentrations were very high in the early years at Rubidoux and declined over time. The change in secondary nitrate concentrations at the LA Main Street site was smaller. The seasonal trend of secondary nitrate is different between sites: it is highest in the winter at Rubidoux and more similar throughout the year at LA Main St. The annual average contribution of diesel vehicles to PM_2.5_ concentrations at LA Main St. and Rubidoux sites showed an increasing trend from 2005 to 2016, although there were some fluctuations, and then decreased from 2017 to 2019. This trend is similar to the PM_2.5_ emission trend from the California Emissions Projection Analysis Model (CEPAM), which projects emissions based on 2017 data. The fractional contribution of diesel vehicles to PM_2.5_ also increased at both sites (from around 13 to 30–40%). The concentration of secondary sulfate was highest in the summer, and the gasoline and diesel vehicle factors were highest in the fall and winter.

The trend of changing dominant PM_2.5_ source factors at the Manhattan, Queens, and Bronx sites is similar (Figure S3). Secondary sulfate was the most important factor in the early years and then decreased over time due to emission controls and the change in generation from coal to natural gas. The next important contributor is secondary nitrate, which has declined over the years. In recent years, the most important source factor for PM_2.5_ at the Manhattan and Bronx sites is gasoline vehicles, while secondary nitrate is the most important for PM_2.5_ at the Queens site. Although biomass burning and residual oil concentrations did not change significantly over time, they represented a larger percentage of the total PM_2.5_ concentrations, as the concentrations of the dominant contributors decreased.

### 3.2 Model Performance and Variable Importance

Since the objective was to understand better the impacts of emission controls on PM_2.5_ concentrations from different sources, we needed to develop models with high accuracy to predict source-apportioned PM_2.5_ concentrations. Most of the predicted daily source-apportioned PM_2.5_ concentrations at each site showed moderate correlations with the built GAMs, with *R*^2^ values ranging from 0.3 to 0.7 (Table 1). An exception is biomass burning, which showed *R*^2^ values of around 0.15–0.4. The simulated PM_2.5_ concentrations from all of the sources tended to be underestimated in all sites. The correlation between observed and predicted daily source-apportioned PM_2.5_ concentrations closely agreed with the performance of the GAM for total PM_2.5_ concentrations except for biomass burning at all sites and diesel vehicles at the New York City sites. Also, the *R*^2^ values for PMF secondary nitrate and secondary sulfate factors at all sites are comparable to, although typically lower than, those of GAMs built for the observed nitrate, sulfate, and ammonium (Table 1).

**Table 1:** Summary of *R*^2^ Values of How Well the GAM Model Reproduced Daily Levels of Each PMF Factor in Atlanta, New York City, and the South Coast Air Basin (SoCAB)

	**Atlanta**	**South Coast Air Basin**		**New York City**		
**sites/sources**	**South Dekalb**	**LA** N Main St.	**Rubidoux**	**Bronx**	**Manhattan**	**Queens**
diesel	0.50	0.55	0.37	0.20	0.21	0.26
gasoline	0.42	0.68	0.59	0.54	0.46	0.33
secondary nitrate	0.58	0.52	0.59	0.45	0.49	0.41
secondary sulfate	0.49	0.61	0.67	0.47	0.55	0.42
soil/road dust	0.28	0.41	0.57	0.30	0.36	0.29
biomass burning	0.20	0.37	0.34	0.16	0.18	0.19
residual oil	NA	NA	NA	0.63	0.34	0.58
total PM_2.5_	0.52	0.40	0.50	0.53	0.47	0.49
observed nitrate	0.53	0.52	0.61	0.54	0.56	0.55
observed sulfate	0.65	0.70	0.70	0.60	0.59	0.60
observed ammonium	0.69	0.60	0.65	0.53	0.50	0.48
observed EC	0.70	0.73	0.57	0.46	0.54	0.53
observed OC	0.51	0.60	0.49	0.54	0.59	0.53

The observed EC and OC concentrations relate to the gasoline and diesel vehicle factors. The model performance of gasoline vehicles and the observed OC concentrations are similar. However, the correlation between the GAM-modeled gasoline vehicles at most sites was worse than that of the observations except for the two sites in the SoCAB. Model performance for fitting diesel vehicle impacts at all sites was much worse than that for the observed EC concentrations. However, the GAMs developed for other sources at each site appear to be effective for estimating source-apportioned PM_2.5_ concentrations overall. The variable importance represents the sensitivity of modeled air pollution concentrations to changes in indicators, which were calculated by differences in correlation values between the observed and the predicted values with the original data set and a version of the data set in which the value of one indicator has been randomly shuffled. The importance of each indicator for each source-apportioned PM_2.5_ is different because of the differences in geography, meteorology, and dominant emission sources (Figure S4).

### 3.3 Impact of Emission Controls on PMF PM_2.5_ Factors

#### South Dekalb, Atlanta, GA

**Figure 2:**
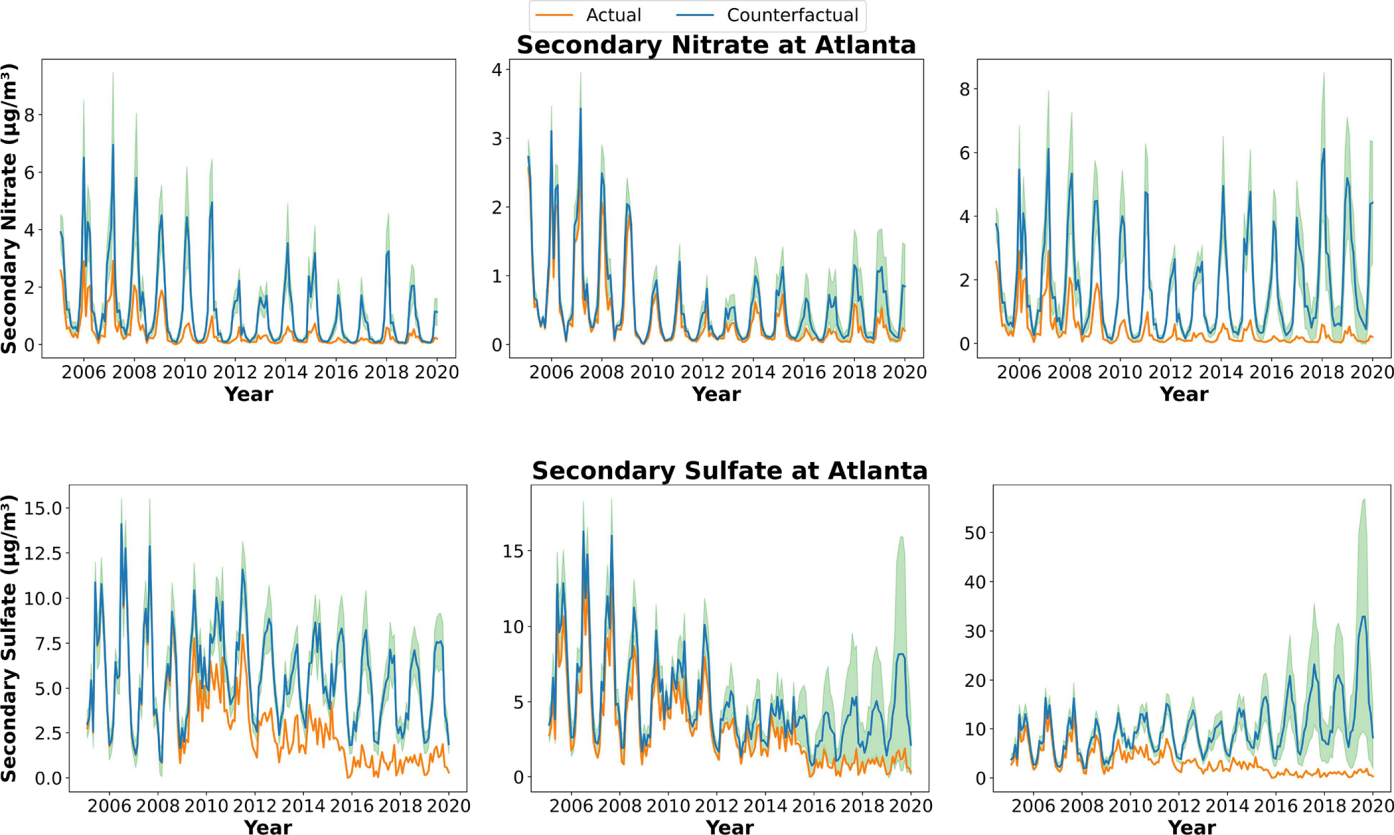
Monthly averaged observations and counterfactual secondary nitrate and sulfate with only counterfactual EGU emissions (left column), only counterfactual mobile emissions (middle column), and total counterfactual emissions (right column) at South Dekalb, GA. The orange line is observed data, and the blue line is counterfactual data. The green area is the uncertainty.

The impact of EGU emission controls on daily concentrations of secondary sulfate and secondary nitrate factors was greater than that of mobile source emission controls in Georgia (Figure 2 and Figure S5). In contrast, mobile emission regulations had a more significant effect on gasoline vehicles, diesel vehicles, and soil and road dust factors. Without EGU emission controls, the annual average secondary sulfate and secondary nitrate concentrations in 2019 would be 4.8 times and 3.4 times higher, respectively, than after the application of these controls (i.e., secondary sulfate would be 4.80 μg/m^3^ compared to 0.99 μg/m^3^, and secondary nitrate would be 0.59 μg/m^3^ compared to 0.17 μg/m^3^). The secondary sulfate factor would have become the dominant contributor to total PM_2.5_ concentrations from 2005 to 2019 without EGU emission controls. Without mobile emission controls, the annual average PM_2.5_ concentrations from diesel vehicles, gasoline vehicles, and soil and road dust would increase by approximately 668, 155, and 411%, respectively (i.e., the diesel vehicles factor would be 5.68 μg/m^3^ compared to 0.85 μg/m^3^, the gasoline vehicles factor would be 4.17 μg/m^3^ compared to 2.69 μg/m^3^, and the soil and road dust factor would be 2.14 μg/m^3^ compared to 0.52 μg/m^3^). Although secondary sulfate was still the leading factor in total PM_2.5_ concentrations in the early years, fractional contributions from gasoline and diesel vehicles have risen. Without both EGU and mobile emission controls, the estimated PM_2.5_ concentrations in 2019 would be 3 times higher than those with the controls. Secondary sulfate would be the leading factor followed by gasoline vehicles, diesel vehicles, and secondary nitrate. The seasonal trend for each factor remains the same, although each factor’s concentrations increase every season. The uncertainty ranges from 7 to 77% without EGU emission controls, from 37 to 89% without mobile emission controls, and from 32 to 78% without all emission controls including the uncertainties in emissions and GAM models. Vehicle-based PM_2.5_ and secondary sulfate have more uncertainties.

#### South Coast Air Basin, California

**Figure 3:**
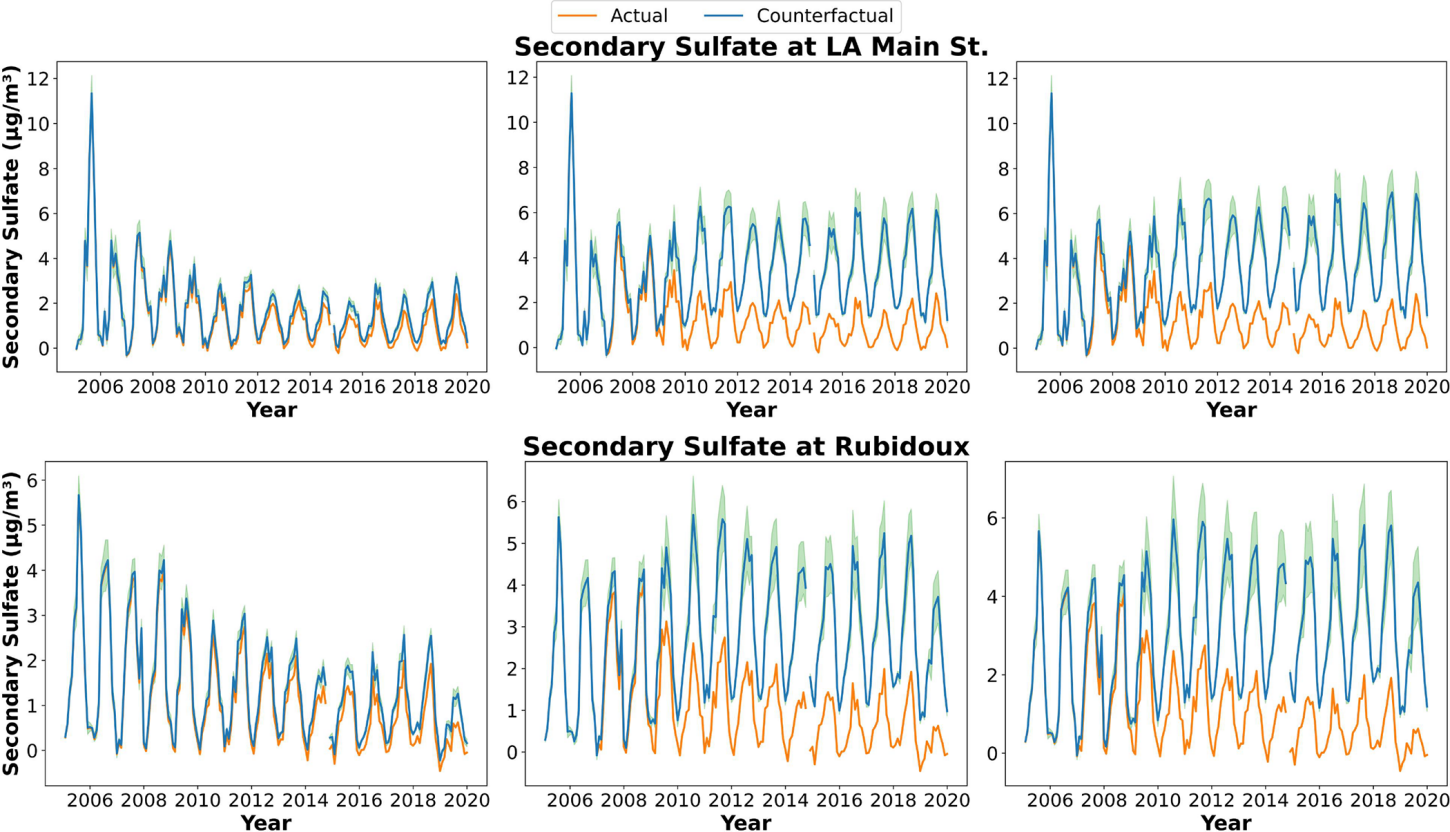
Monthly averaged observations and counterfactual secondary sulfate with only counterfactual on-road emissions (left column), only counterfactual port emissions (middle column), and total counterfactual emissions (right column) at LA Main St. (top) and Rubidoux (bottom), CA. The orange line is observed data, and the blue line is counterfactual data. The green area is the uncertainty.

The effect of on-road emission controls on daily PM_2.5_ concentrations was observed in most sources except secondary sulfate, which was significantly affected by port regulations in SoCAB (Figure 3 and Figure S6). In 2019, without control policies at the LA Main St. and [Rubidoux] sites, the annual average concentrations of PM_2.5_ from secondary nitrate, gasoline vehicles, and diesel vehicles would be 9.05 [9.91], 4.95 [2.07], and 7.65 μg/m^3^ [4.60 μg/m^3^], which is 3.3 [3.2], 7.7 [2.5], and 4.4 [1.4] times higher, respectively, than the scenario with emissions controls (the annual average was 2.72 [3.13], 0.64 [0.84], and 1.75 μg/m^3^ [3.35 μg/m^3^] with emission controls; the values in parentheses are the concentrations at Rubidoux). The concentration of the secondary sulfate factor would be approximately 3.8 and 12 times higher (from 0.86 to 3.30 μg/m^3^ at the LA Main St. site and from 0.18 to 2.18 μg/m^3^ at Rubidoux) without port emission regulations in 2019. Without both on-road and port emission controls, the estimated annual PM_2.5_ concentrations (the sum of all the factors) in 2019 would be 4.5 and 2.8 times higher than with the controls at the LA Main St. and Rubidoux sites (from 6.62 to 29.5 μg/m^3^ at the LA Main St. site and from 8.91 to 25.1 μg/m^3^ at Rubidoux). The secondary nitrate factor would become the most dominant contributor to total PM_2.5_ concentrations from 2005 to 2019 without on-road or port emission controls. However, the secondary sulfate factor would be the second leading contributor without port emission controls, and the actual concentration value and fractional contribution to total PM_2.5_ concentration would be very close to those of the secondary nitrate factor in 2019. The seasonal trend for each factor would remain the same, although the concentrations for each factor would increase in every season. The uncertainty ranges from 2.7 to 36% [11 to 122%] without port emission controls, from 15 to 147% [5 to 68%] without on-road emission controls, and from 12 to 135% [11 to 270%] without all mobile sources emission controls at the LA Main St. site [Rubidoux site] including the uncertainties in emissions and GAM models. PM_2.5_ from vehicles and biomass burning have more uncertainties.

#### Bronx, Manhattan, and Queens, New York

**Figure 4:**
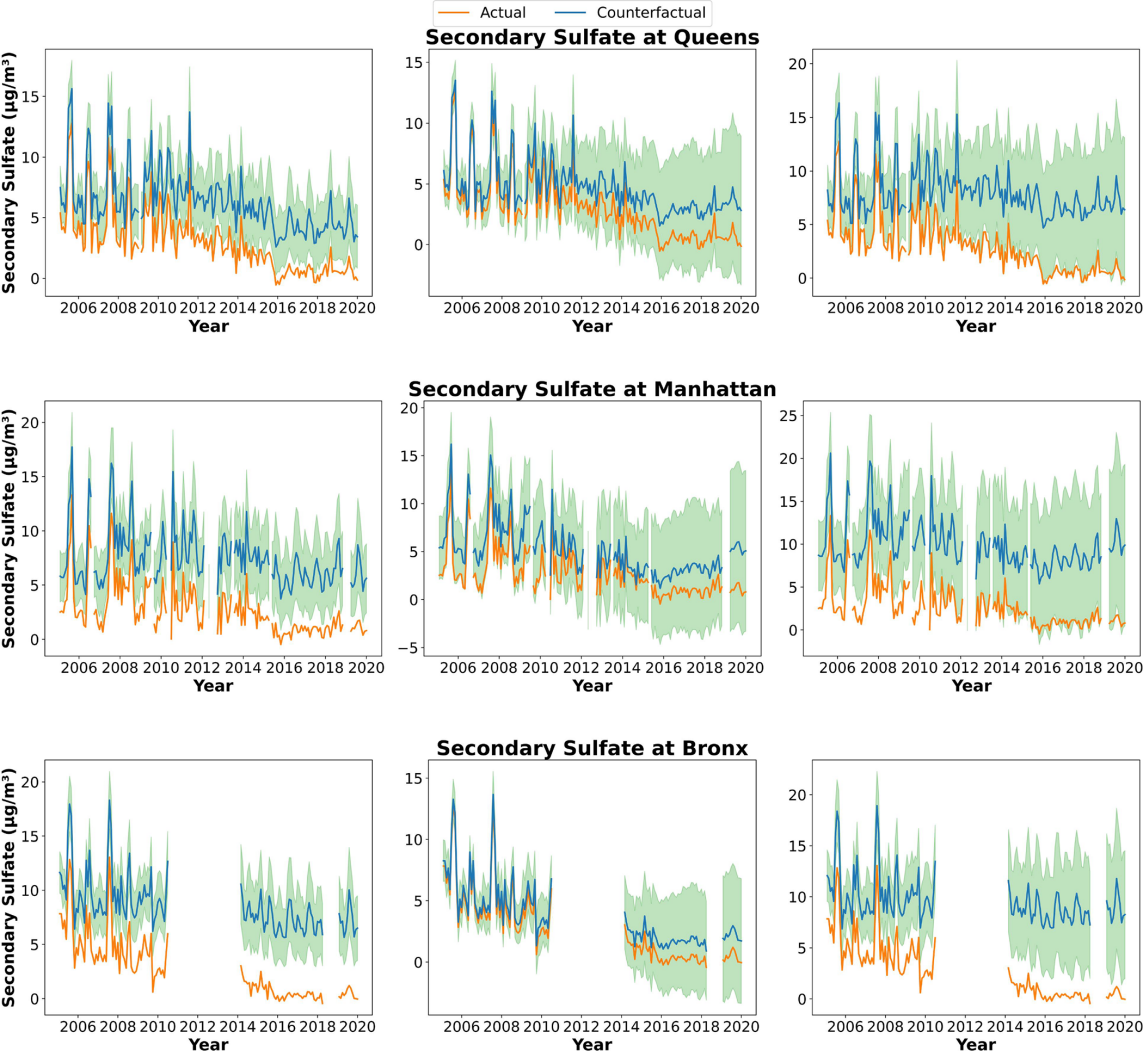
Monthly averaged observations and counterfactual air pollution levels with only counterfactual EGU emissions (left column), only counterfactual mobile emissions (middle column), and total counterfactual emissions (right column) at Queens (top), Manhattan (middle), and Bronx (bottom), NY. The orange line is observed data, and the blue line is counterfactual air quality data. The green area is the uncertainty.

The changes in emissions from EGUs on daily PM_2.5_ concentrations were primarily observed in the secondary nitrate and secondary sulfate factors (Figure 4 and Figure S7). In the absence of those changes, the annual average PM_2.5_ concentrations from secondary nitrate in 2019 would be approximately 3.9 and 2.3 times higher in Queens and the Bronx, respectively, and secondary sulfate would be 5.7 and 8.1 times higher at Manhattan and Queens (secondary nitrate: from 0.53 to 4.26 μg/m^3^ at the Manhattan site, from 1.63 to 6.30 μg/m^3^ at the Queens site, and from 1.63 to 3.77 μg/m^3^ at the Bronx site; secondary sulfate: from 1.03 to 5.89 μg/m^3^ at the Manhattan site, from 0.52 to 4.22 μg/m^3^ at the Queens site, and from 0.42 to 7.18 μg/m^3^ at the Bronx site), respectively. EGU controls had the largest impact on the secondary nitrate factor at the Manhattan site, where concentrations would be around 8 times higher, and on the secondary sulfate factor at the Bronx site, where concentrations would be about 17 times higher without EGU emission controls. Consequently, the leading factor at the Manhattan site from 2005 to 2019 would be secondary sulfate; at the Queens site, it would be secondary nitrate; and at the Bronx site, the leading factor would shift from secondary nitrate to secondary sulfate without EGU control policies.

The counterfactual daily PM_2.5_ concentrations without mobile control policies from secondary sulfate, secondary nitrate, gasoline vehicles, and diesel vehicles would be 5.30, 11.1, 4.05, and 3.26 μg/m^3^, which are about 5.1, 21, 2.6, and 3.6 times higher, respectively, than the actual daily source-apportioned PM_2.5_ concentrations at Manhattan in 2019 (1.03, 0.53, 1.53, and 0.90 μg/m^3^). The mobile control policies had a significant impact on secondary nitrate concentrations at the Manhattan site, which has the largest difference between counterfactuals and estimated source-apportioned concentrations. The secondary sulfate, secondary nitrate, and soil and road dust factors at the Queens site are affected by mobile control policies significantly (secondary sulfate is from 0.52 (actual) to 3.47 μg/m^3^ (counterfactual) [6.7 times], secondary nitrate is from 1.63 (actual) to 14 μg/m^3^ (counterfactual) [8.7 times], and soil and road dust is from 0.06 (actual) to 0.64 μg/m^3^ (counterfactual) [10 times, which is due to primary PM_2.5_ emissions from mobile source (Figure S2) in 2019]. Other factors slightly increase without emission controls compared to these three factors (an increase of around 2.3 times). The significantly increased factors at the Bronx site were secondary sulfate and soil and road dust (both 5 times higher: secondary sulfate is from 0.42 to 2.18 μg/m^3^, and soil and road dust is from 0.29 to 1.48 μg/m^3^). Consequently, the leading factor at Queens was secondary nitrate from 2005 to 2019, indicating that mobile control policies had a greater impact on the leading factor than EGU control policies at Queens. At the Manhattan and Bronx sites, the leading factor shifted from secondary sulfate to secondary nitrate, suggesting that EGU control policies had a significant impact on the leading factors at both the Bronx and Manhattan sites.

Without any emissions controls, the annual average simulated PM_2.5_ concentrations (sum of all source-specific PM_2.5_ concentrations) in 2019 would be 6 times higher than with the concentrations after controls at the Manhattan site, approximately 7 times higher at the Queens sites, and 3.5 times higher at the Bronx site in New York City compared to when controls are applied (Manhattan: from 5.25 to 31.5 μg/m^3^; Queens: 4.67 to 31.2 μg/m^3^; Bronx: 6.25 to 21.8 μg/m^3^). The secondary sulfate factor would become the most dominant contributor to total PM_2.5_ concentrations from 2005 to 2019 at the Bronx and Manhattan without these emission controls, while the secondary nitrate factor would be the leading contributor at Queens. The seasonal trend for each factor remained constant, although the concentrations for each factor increased in each season. The uncertainty ranges from 8.9 to 77% [19 to 280%; 1.8 to 203%] without EGU emission controls, from 8.9 to 172% [36 to 296%; 21 to 278%] without mobile emission controls, and from 8.9 to 82% [36 to 296%; 21 to 224%] without all emission controls at the Manhattan site [Queens and Bronx sites] including the uncertainties in emissions and GAM models. PM_2.5_ from secondary inorganics, vehicles, and soil/road dust has more uncertainties. PM_2.5_ concentrations fluctuated seasonally with noticeable peaks during the colder months. The large green area represents the uncertainties due to the relatively poorer fit between the GAM model for the New York sites versus the other locations and as compared to individual species.^17^

The trends in the observed total PM_2.5_ concentrations are similar to those of the source-apportioned PM_2.5_ concentrations across all sites. However, at most sites, except for Queens, the observed PM_2.5_ exceeded the combined contributions most of the time (Figure S3). Both observed PM_2.5_ and combined source-apportioned PM_2.5_ concentrations showed an overall decreasing trend with some fluctuations, likely due to emission controls and economic drivers.^21,41^

**Figure 5:**
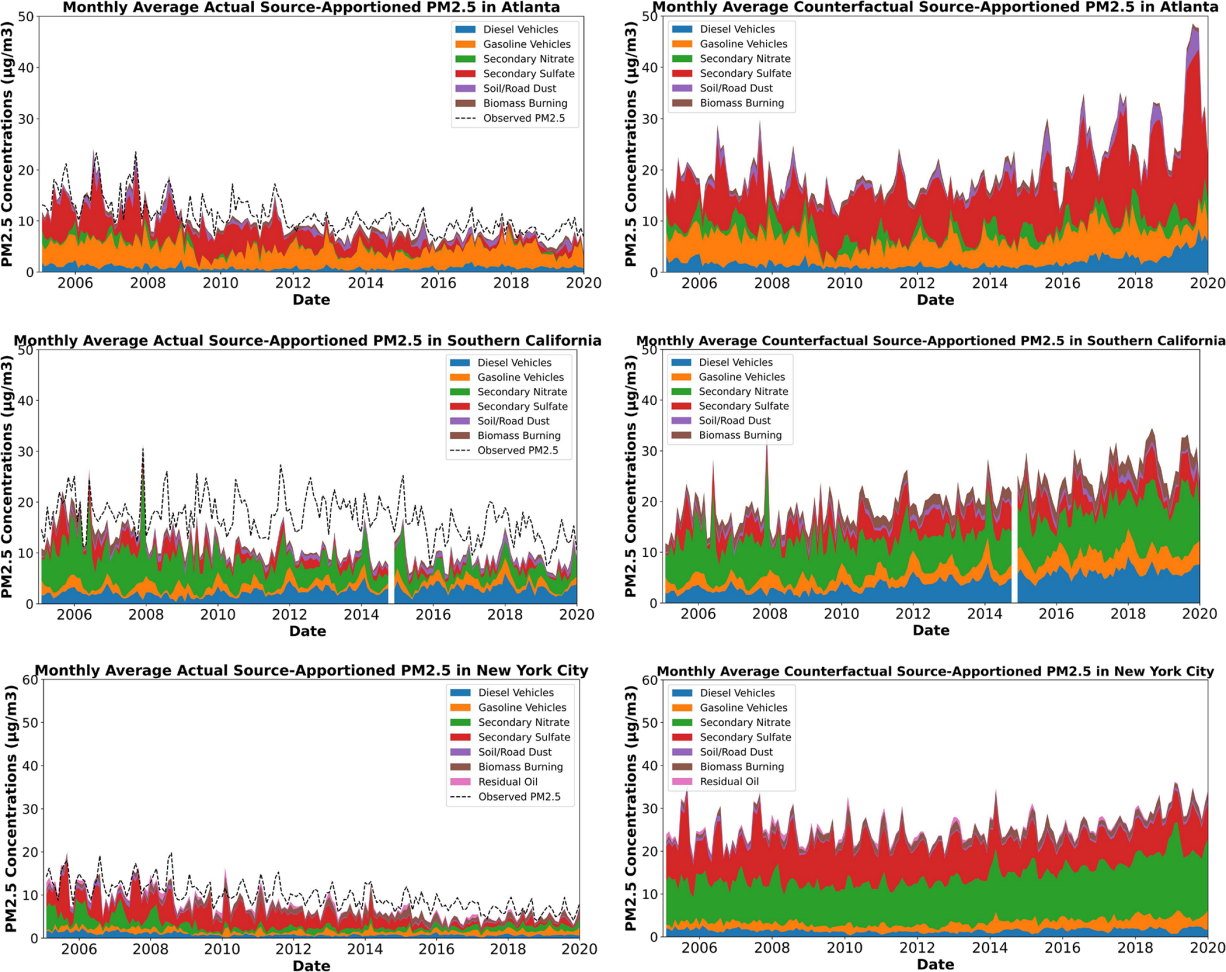
Monthly averaged observations (left column) and counterfactual source-apportioned PM_2.5_ levels with total counterfactual emissions (right column) at Atlanta, South Coast Air Basin (averaged concentrations at LA Main Street and Rubidoux), and New York City (averaged concentrations at Queens and Manhattan). The blue, orange, green, red, purple, brown, and pink areas represent the PM_2.5_ concentrations from diesel vehicles, gasoline vehicles, secondary nitrate, secondary sulfate, soil/road dust, biomass burning, and residual oil. The black dashed line shows the observed total PM_2.5_ concentrations. Each site’s results are shown in Figure S3.

Compared with the observed source-apportioned PM_2.5_ concentrations, the combined counterfactual source-apportioned PM_2.5_ concentrations, without emission controls, are significantly higher (Figure 5 and Figure S3). The counterfactual source-apportioned PM_2.5_ concentrations show an increasing trend, or they remained flat, suggesting that emission controls had a significant impact on mitigating PM_2.5_ concentrations from these sources. In addition, while the seasonal trends are consistent with the observations, the peaks in PM_2.5_ are sharper and more pronounced in the counterfactual scenario, indicating the influence of emission controls on seasonal air quality fluctuations.

## 4 Discussion

The GAMs for source-apportioned PM_2.5_ concentrations effectively captured the observed daily fluctuations in the contributions of various factors. GAM modeling demonstrated slightly greater accuracy in simulating both total PM_2.5_ and its individual species rather than the individual source factors themselves (Table 1). This result is seen when comparing the model performance of these GAMs with the observed total PM_2.5_ concentrations and specific PM_2.5_ species concentrations in our previous analyses.^35,42^

In Georgia, EGU emission controls significantly affected secondary nitrate and secondary sulfate, two key components of PM_2.5_. The contributions of observed secondary nitrate and secondary sulfate accounted for about 50% of the total PM_2.5_ concentrations in 2005 but decreased to 20% by 2019. Secondary inorganic aerosol concentrations increased from 1.16 μg/m^3^ with EGU emission controls to 5.38 μg/m^3^ without EGU policies in 2019, roughly 5-fold higher, with their contribution to PM_2.5_ reaching around 45%, similar to that in 2005. The higher proportion of secondary inorganic PM_2.5_ within the total PM_2.5_ concentrations in the absence of EGU emission controls suggests that EGU emission controls effectively reduced the level of PM_2.5_. Mobile emission controls mainly affected the contributions of gasoline and diesel vehicles to PM_2.5_. PM_2.5_ concentrations from vehicles increased from 3.54 to 9.85 μg/m^3^ in scenarios without mobile emission controls, nearly tripling in concentrations. The increase in vehicle-derived PM_2.5_ concentrations without mobile emission controls indicates that mobile emission controls successfully mitigated the PM_2.5_ produced by vehicles. Compared to the actual combined source-apportioned PM_2.5_, the counterfactual PM_2.5_ without regulations peaked at 35.1 μg/m^3^ by 2019, nearly 6 times the actual concentration of 5.77 μg/m^3^. Also, the dominant factor in the actual scenario shifted from secondary sulfate to gasoline vehicles over time, whereas secondary sulfate would consistently remain the dominant contributor in the counterfactual scenario.

Changes in EGUs were more effective at reducing secondary nitrate and secondary sulfate concentrations in New York compared to those in Georgia. This may be due to the benefits of EGU controls on upwind emissions originating from the Ohio River Valley and the midwestern states. The dominant contributor shifted from secondary sulfate to gasoline vehicles with EGU emission controls, whereas secondary inorganic aerosols remained the dominant contributor in the counterfactual scenario. The contribution of secondary inorganic aerosols to PM_2.5_ would be approximately 5.5 times higher compared to the actual contributions (increasing from 1.92 to 10.5 μg/m^3^ in New York City, averaging across the three sites), and the proportion of secondary inorganic aerosols would shift from 36% in the actual scenario to 75% without EGU regulations. In addition to EGU emission controls, mobile emission controls also significantly affect PM_2.5_ from secondary nitrate (in Manhattan and Queens) or secondary sulfate (in the Bronx). In the absence of the mobile source control policies, the contribution of secondary nitrate to PM_2.5_ would increase from 1.08 to 12.6 μg/m^3^ (approximately 12 times, averaging Queens and Manhattan), and the percentage in the combined source-apportioned PM_2.5_ would increase from 22 to 51%. This result suggests that mobile emission controls not only directly reduced primary emissions but also limited the availability of NH_3_ for secondary nitrate formation, mitigating its overall contribution to PM_2.5_. The secondary sulfate contribution in the Bronx would increase from 0.42 to 2.27 μg/m^3^ (about 5.4 times), and the percentage would rise from 6.8 to 17%. Moreover, mobile emission controls reduce the contribution of soil and road dust to PM_2.5_ in New York, with the contribution increasing from 0.20 to 0.91 μg/m^3^ (about 4.5 times) without controls. In Queens and Manhattan, mobile emission controls have the most significant influence on reducing PM_2.5_ concentrations, with an impact 1.8 times greater than that of EGU emission controls, likely due to the high traffic density in these areas. Conversely, in the Bronx, EGU emission controls are more significant in reducing PM_2.5_, with an impact 1.2 times greater than that of mobile emission controls, as this site may be more affected by emissions from power plants.

Port regulations had significant impacts on the reduction of secondary sulfate, especially at Rubidoux, mainly because ports are major sources of sulfur dioxide (SO_2_), a precursor to secondary sulfate. This reduction is also intertwined with heavy-duty diesel regulations, which reduce SO_2_ emissions from diesel trucks and trains. The difference in the effectiveness of port emission controls between these two sites suggests that geographic distance to emission sources can influence emission controls. Secondary sulfate concentrations increased from 0.18 to 2.18 μg/m^3^ at Rubidoux, and its contribution to the combined source-apportioned PM_2.5_ rose from 2 to 15%. Secondary sulfate increased from 0.86 to 3.30 μg/m^3^ at LA Main St., about a 4-fold increase, and the contribution to the combined source-apportioned PM_2.5_ changed from 13 to 30%. The dominant contributor to actual PM_2.5_ in Southern California was secondary nitrate, whereas secondary sulfate can compete with secondary nitrate in the counterfactual scenario. On-road emission regulations had a more significant impact on PM_2.5_ from gasoline and diesel vehicles and secondary nitrate formation at the LA Main Street site compared to Rubidoux. Contributions from vehicle emissions and secondary nitrate increased from 5.11 to 21.6 μg/m^3^ at the LA Main St. site (∼4 times higher), raising the contribution to the combined source-apportioned PM_2.5_ from 77 to 86%. These factors increased from 7.32 to 16.6 μg/m^3^, doubling in concentration, and their contribution rose from 82 to 86%. The formation of secondary nitrate is through the oxidation of nitrogen oxides (NOx), the primary air pollutants from mobile emissions. The heavier traffic volume near the LA Main St. site may be the reason for the greater reduction in secondary nitrate compared to the Rubidoux site. Also, the super ultra-low emission vehicles (SULEVs) were introduced in California in 2015, whereas they were not implemented until 2017 in Georgia or New York. LA Main Street and New York City results suggest that on-road emission controls are important in controlling secondary nitrate PM_2.5_, especially in urban areas with significant vehicular activity.

There are several limitations in this study. First, a key limitation is that the PMF model does not explicitly account for the nonlinear chemical interactions involved in secondary PM_2.5_ formation. Second, the GAM framework allows for flexible, nonlinear relationships between emissions, meteorology, and source-apportioned PM_2.5_ concentrations. However, it may not fully capture how changes in precursor emissions influence secondary aerosol formation under varying chemical regimes as it does not include all potential interactions between indicators. Although we included some interaction terms between emissions and meteorology as well as between different emissions sources manually, some complex dependencies may still be missing. Finally, we used annual emissions data to build models with categorical variables (e.g., day of the week and day of the year) to represent temporal variations in emissions. The accuracy of counterfactual concentrations could be improved if daily emission estimates for each sector were available, allowing for a more precise representation of short-term variations in emission patterns.In summary, the influence of emission controls on PM_2.5_ concentrations from different sources varied substantially based on major emission sources and geographical location. The implementation of various emission control policies has the potential to shift the dominant contributing factor to PM_2.5_ concentrations. Total PM_2.5_ concentrations could increase by as much as 3 to 7 times without these emission controls. While the seasonal trends for each factor remained consistent over time, the PM_2.5_ concentrations from each factor increased across all seasons.

## Supporting Information Available

Changes in air pollutant emissions from EGU and mobile sources in Georgia, New York, and the South Coast Air Basin; monthly averaged actual and counterfactual source-apportioned PM_2.5_ concentrations; the variable importance of each indicator in source-apportioned PM_2.5_ GAMs; a flowchart of Section 2.2.1; and the set of indicators used in each PMF factor’s GAM (PDF)
