# Supplementary material for: Accountability Assessment of Source-Specific Impacts of Regulations on Emissions and Air Quality Using Positive Matrix Factorization
Source: Environ Sci Technol. 2025 Apr 24;59(17):8651–61. doi: 10.1021/acs.est.4c12511 (PMC12060267; doi:10.1021/acs.est.4c12511)
Supplement: Supplementary file 1 — es4c12511_si_001.pdf [file es4c12511_si_001.pdf]

Supplementary Information

Accountability assessment of source-specific impacts of regulations on emissions and air quality using Positive Matrix Factorization

Ziqi Gao,[1,*,#] Eric J. Mei,[1,##] Xin He,[1] Stefanie Ebelt,[3] David Q. Rich,[2] Armistead G. Russell[1]

[1] School of Civil and Environmental Engineering, Georgia Institute of Technology, Atlanta, GA 30332, United States

[2] Department of Public Health Sciences, University of Rochester School of Medicine and Dentistry, Rochester, NY 14642, United States

[3] Gangarosa Department of Environmental Health, Rollins School of Public Health, Emory University, Atlanta, GA 30322, United States

[#] Currently: University of Virginia Environmental Institute, Charlottesville, VA 22902, United States

[##] Currently: Department of Atmospheric and Climate Science, University of Washington, Seattle, WA 98195, United States

* Email: zgao71@gatech.edu

SI includes: 18 pages, 8 figures, and 2 tables

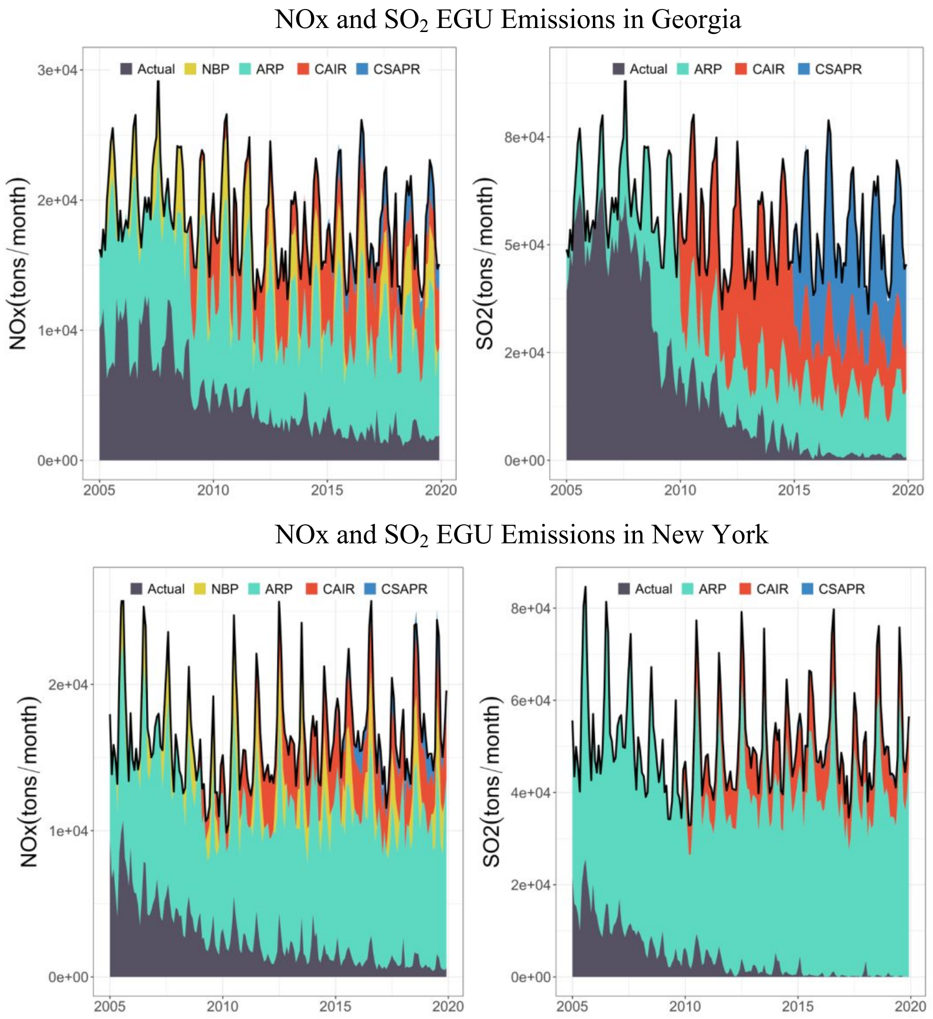

Figure S1. Change in NOx and SO_2 emissions from EGU sources in Georgia (top) and New York (bottom). The grey area shows the reported (actual) emissions, the yellow area is the modeled impact of the NOx SIP call and NBP, green is ARP, red is CAIR, blue is CSAPR, and the black line shows the counterfactual EGU emissions (Adapted from [1]. Available under a CC-BY 4.0. Copyright 2024 Elsevier).

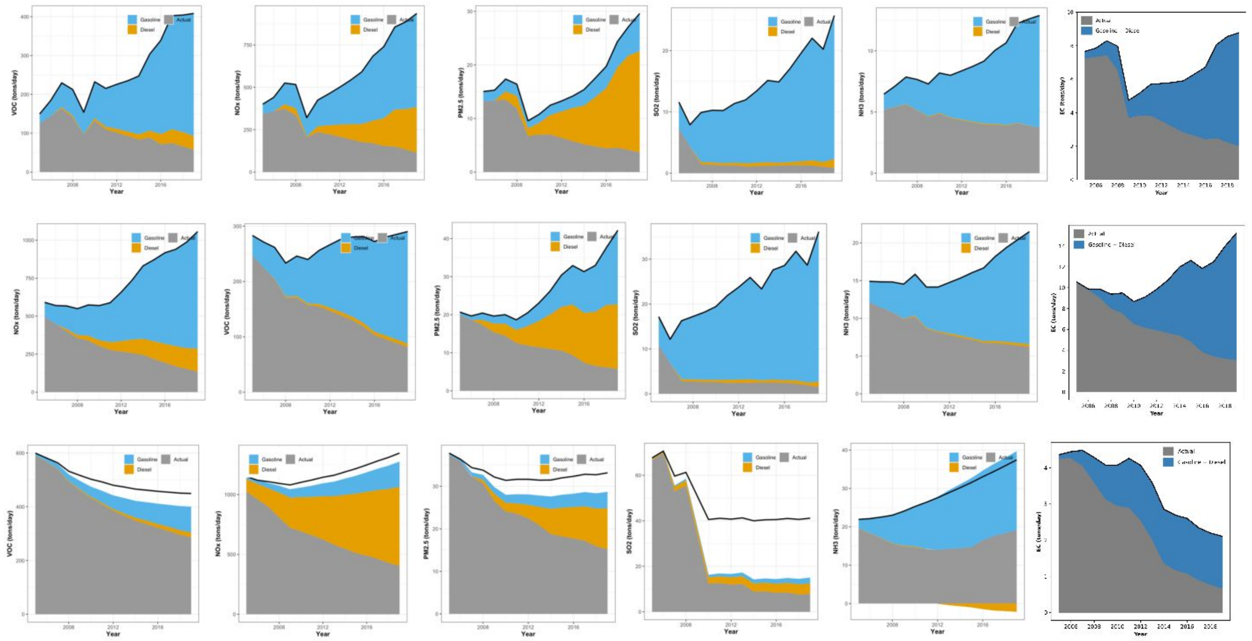

Figure S2. The change in VOC, NOx, $PM_{2.5}$, SO_2, NH_3, and EC emissions from mobile sources due to different emissions regulations in Georgia (top), New York (middle), and South Coast Air Basin (bottom). The grey area shows the reported (actual) emissions, the yellow area is the diesel program, the blue area is the gasoline program, the dark blue area is the gasoline and diesel programs for EC emissions, the blank area shows the port emission controls, and the black line shows the counterfactual mobile emissions (Adapted from [1]. Available under a CC-BY 4.0. Copyright 2024 Elsevier).

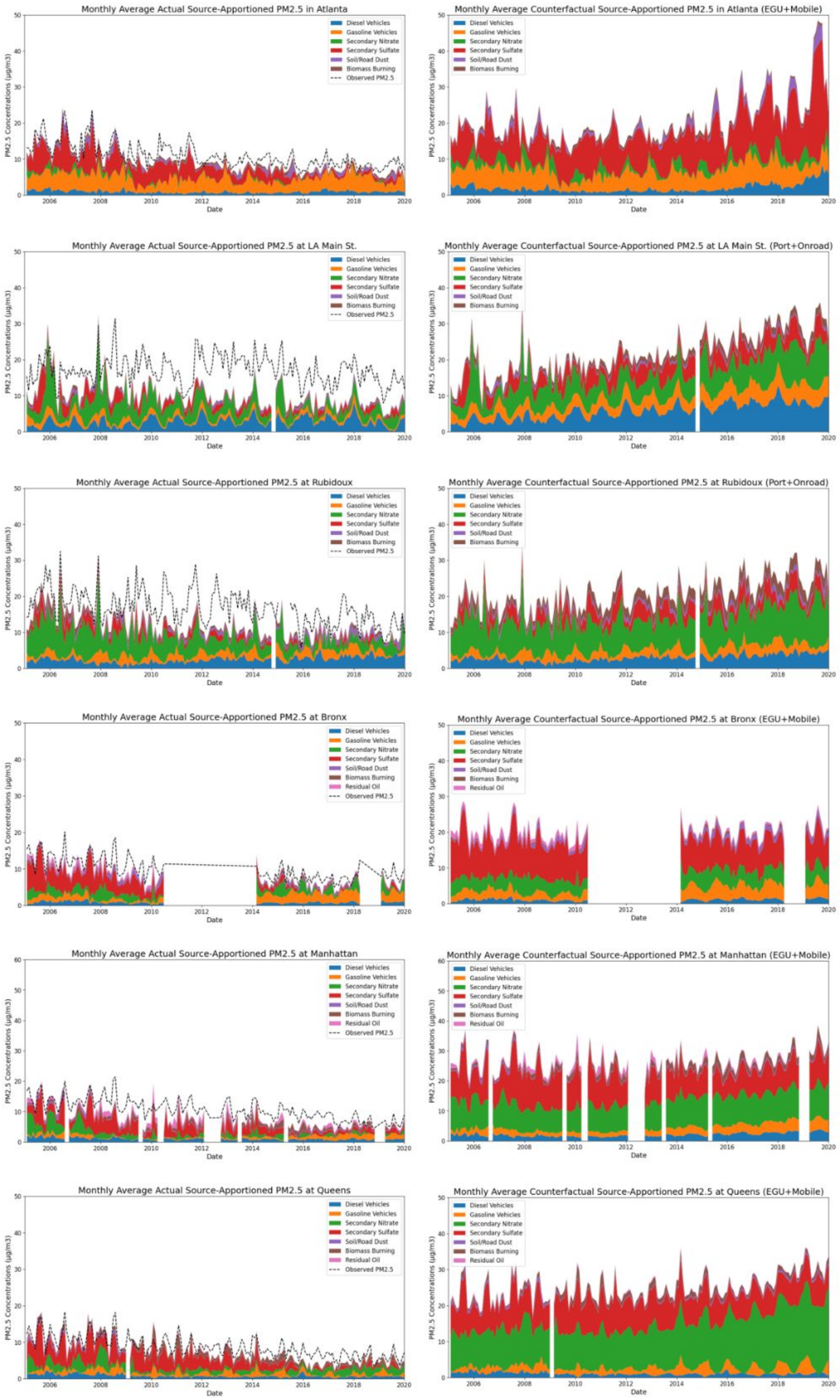

Figure S3. Monthly averaged observations (left column) and counterfactual source apportioned $PM_{2.5}$ levels with total counterfactual emissions (right column) at South Dekalb, GA, LA Main St. and Rubidoux, California, and Manhattan, Queens and Bronx, New York. The blue, orange, green, red, purple, brown and pink areas represent the $PM_{2.5}$ concentrations from diesel vehicles, gasoline vehicles, secondary nitrate, secondary sulfate, soil/road dust, biomass burning, and residual oil. The black dash line shows the observed total $PM_{2.5}$ concentrations.

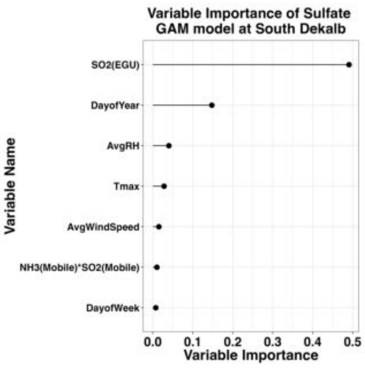
Variable Importance of Sulfate
GAM model at South Dekalb
SO2(EGU)
DayofYear
AvgRH
Tmax
AvgWindSpeed
NH3(Mobile)*SO2(Mobile)
DayofWeek
Variable Name
0.0 0.1 0.2 0.3 0.4 0.5
Variable Importance

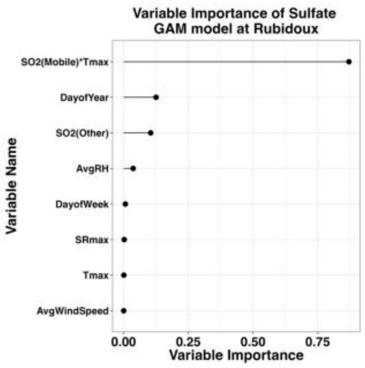
Variable Importance of Sulfate
GAM model at Rubidoux
SO2(Mobile)*Tmax
DayofYear
SO2(Other)
AvgRH
DayofWeek
SRmax
Tmax
AvgWindSpeed
Variable Name
0.00 0.25 0.50 0.75
Variable Importance

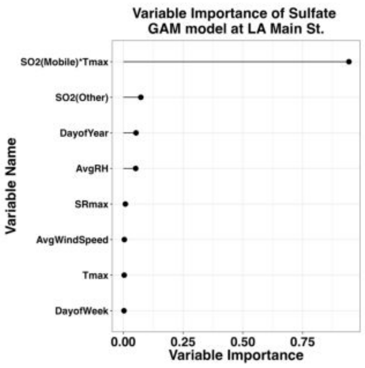
Variable Importance of Sulfate
GAM model at LA Main St.
SO2(Mobile)*Tmax
SO2(Other)
DayofYear
AvgRH
SRmax
AvgWindSpeed
Tmax
DayofWeek
Variable Name
0.00 0.25 0.50 0.75
Variable Importance

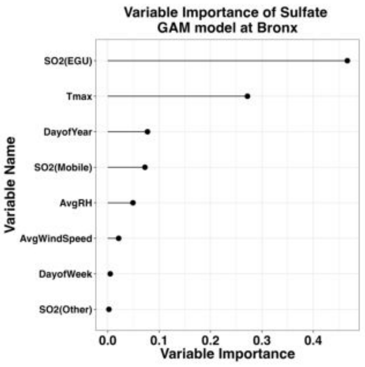
Variable Importance of Sulfate
GAM model at Bronx
SO2(EGU)
Tmax
DayofYear
SO2(Mobile)
AvgRH
AvgWindSpeed
DayofWeek
SO2(Other)
Variable Name
0.0 0.1 0.2 0.3 0.4
Variable Importance

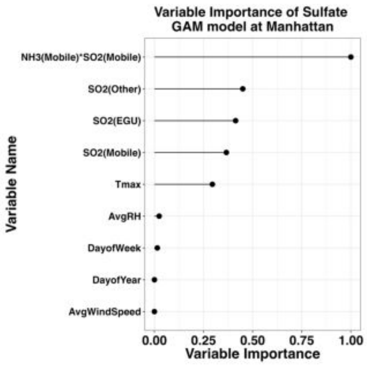
Variable Importance of Sulfate
GAM model at Manhattan
NH3(Mobile)*SO2(Mobile)
SO2(Other)
SO2(EGU)
SO2(Mobile)
Tmax
AvgRH
DayofWeek
DayofYear
AvgWindSpeed
Variable Name
0.00 0.25 0.50 0.75 1.00
Variable Importance

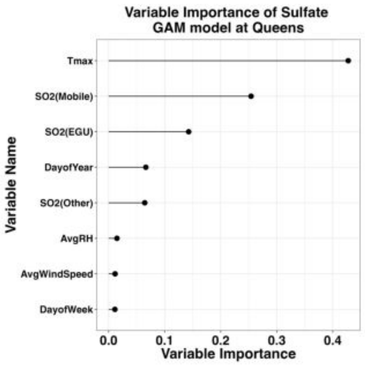
Variable Importance of Sulfate
GAM model at Queens
Tmax
SO2(Mobile)
SO2(EGU)
DayofYear
SO2(Other)
AvgRH
AvgWindSpeed
DayofWeek
Variable Name
0.0 0.1 0.2 0.3 0.4
Variable Importance

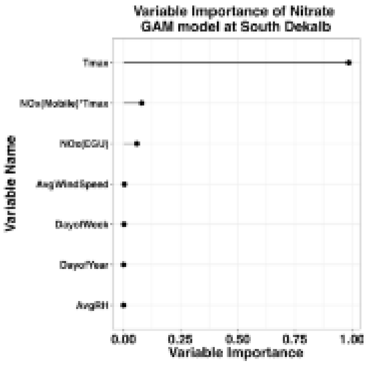
Variable Importance of Nitrate
GAM model at South Dekalb
Tmax
NOx(Mobile)*Tmax
NOx(EGU)
AvgWindSpeed
DayofWeek
DayofYear
AvgRH
Variable Name
0.00 0.25 0.50 0.75 1.00
Variable Importance

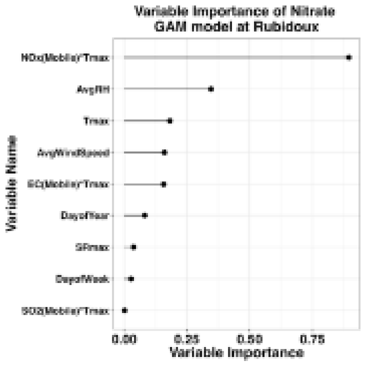
Variable Importance of Nitrate
GAM model at Rubidoux
NOx(Mobile)*Tmax
AvgRH
Tmax
AvgWindSpeed
EC(Mobile)*Tmax
DayofYear
SRmax
DayofWeek
SO2(Mobile)*Tmax
Variable Name
0.00 0.25 0.50 0.75
Variable Importance

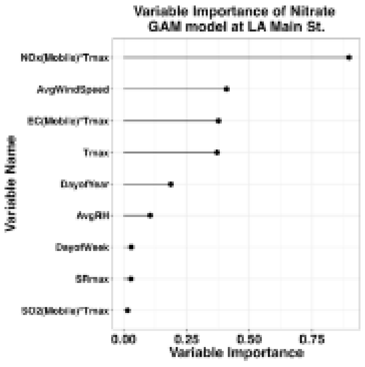
Variable Importance of Nitrate
GAM model at LA Main St.
NOx(Mobile)*Tmax
AvgWindSpeed
EC(Mobile)*Tmax
Tmax
DayofYear
AvgRH
DayofWeek
SRmax
SO2(Mobile)*Tmax
Variable Name
0.00 0.25 0.50 0.75
Variable Importance

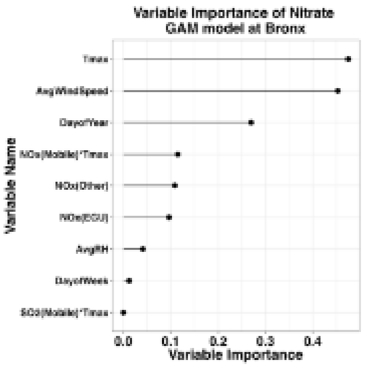
Variable Importance of Nitrate
GAM model at Bronx
Tmax
AvgWindSpeed
DayofYear
NOx(Mobile)*Tmax
NOx(Other)
NOx(EGU)
AvgRH
DayofWeek
SO2(Mobile)*Tmax
Variable Name
0.0 0.1 0.2 0.3 0.4
Variable Importance

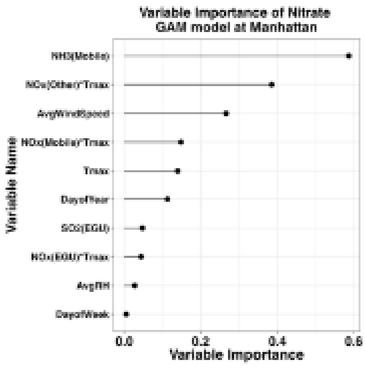
Variable Importance of Nitrate
GAM model at Manhattan
NH3(Mobile)
NOx(Other)*Tmax
AvgWindSpeed
NOx(Mobile)*Tmax
Tmax
DayofYear
SO2(EGU)
NOx(EGU)*Tmax
AvgRH
DayofWeek
Variable Name
0.0 0.2 0.4 0.6
Variable Importance

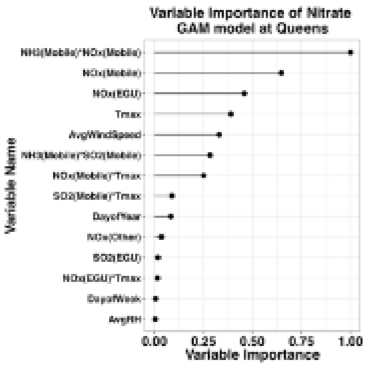
Variable Importance of Nitrate
GAM model at Queens
NH3(Mobile)*NOx(Mobile)
NOx(Mobile)
NOx(EGU)
Tmax
AvgWindSpeed
NH3(Mobile)*SO2(Mobile)
NOx(Mobile)*Tmax
SO2(Mobile)*Tmax
DayofYear
NOx(Other)
SO2(EGU)
NOx(EGU)*Tmax
DayofWeek
AvgRH
Variable Name
0.00 0.25 0.50 0.75 1.00
Variable Importance

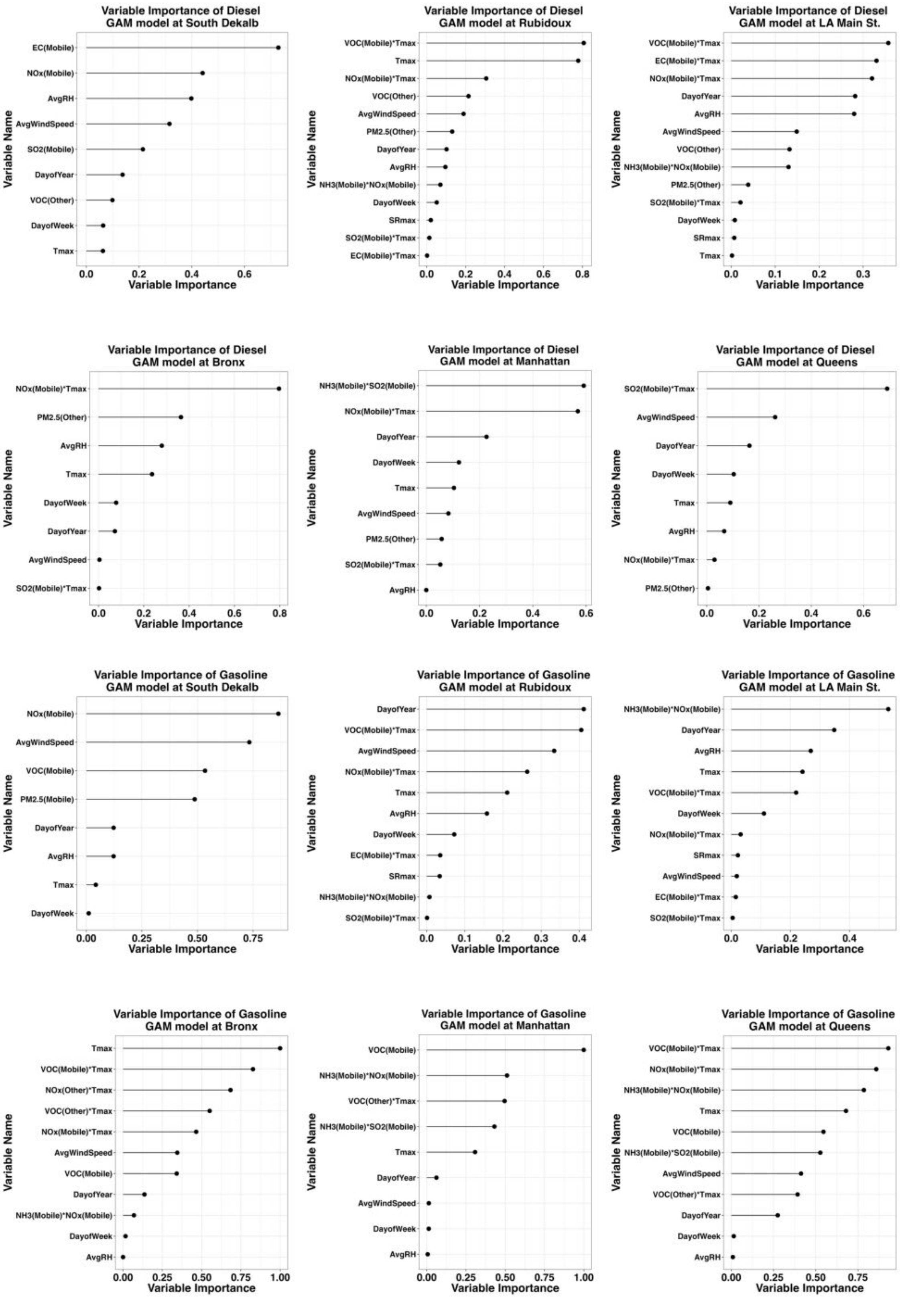
Variable Importance of Diesel GAM model at South Dekalb
EC(Mobile)
NOx(Mobile)
AvgRH
AvgWindSpeed
SO2(Mobile)
DayofYear
VOC(Other)
DayofWeek
Tmax
Variable Name
Variable Importance
0.0 0.2 0.4 0.6
Variable Importance of Diesel GAM model at Rubidoux
VOC(Mobile)*Tmax
Tmax
NOx(Mobile)*Tmax
VOC(Other)
AvgWindSpeed
PM2.5(Other)
DayofYear
AvgRH
NH3(Mobile)*NOx(Mobile)
DayofWeek
SRmax
SO2(Mobile)*Tmax
EC(Mobile)*Tmax
0.0 0.2 0.4 0.6 0.8
Variable Importance of Diesel GAM model at LA Main St.
VOC(Mobile)*Tmax
EC(Mobile)*Tmax
NOx(Mobile)*Tmax
DayofYear
AvgRH
AvgWindSpeed
VOC(Other)
NH3(Mobile)*NOx(Mobile)
PM2.5(Other)
SO2(Mobile)*Tmax
DayofWeek
SRmax
Tmax
0.0 0.1 0.2 0.3
Variable Importance of Diesel GAM model at Bronx
NOx(Mobile)*Tmax
PM2.5(Other)
AvgRH
Tmax
DayofWeek
DayofYear
AvgWindSpeed
SO2(Mobile)*Tmax
0.0 0.2 0.4 0.6 0.8
Variable Importance of Diesel GAM model at Manhattan
NH3(Mobile)*SO2(Mobile)
NOx(Mobile)*Tmax
DayofYear
DayofWeek
Tmax
AvgWindSpeed
PM2.5(Other)
SO2(Mobile)*Tmax
AvgRH
0.0 0.2 0.4 0.6
Variable Importance of Diesel GAM model at Queens
SO2(Mobile)*Tmax
AvgWindSpeed
DayofYear
DayofWeek
Tmax
AvgRH
NOx(Mobile)*Tmax
PM2.5(Other)
0.0 0.2 0.4 0.6
Variable Importance of Gasoline GAM model at South Dekalb
NOx(Mobile)
AvgWindSpeed
VOC(Mobile)
PM2.5(Mobile)
DayofYear
AvgRH
Tmax
DayofWeek
0.00 0.25 0.50 0.75
Variable Importance of Gasoline GAM model at Rubidoux
DayofYear
VOC(Mobile)*Tmax
AvgWindSpeed
NOx(Mobile)*Tmax
Tmax
AvgRH
DayofWeek
EC(Mobile)*Tmax
SRmax
NH3(Mobile)*NOx(Mobile)
SO2(Mobile)*Tmax
0.0 0.1 0.2 0.3 0.4
Variable Importance of Gasoline GAM model at LA Main St.
NH3(Mobile)*NOx(Mobile)
DayofYear
AvgRH
Tmax
VOC(Mobile)*Tmax
DayofWeek
NOx(Mobile)*Tmax
SRmax
AvgWindSpeed
EC(Mobile)*Tmax
SO2(Mobile)*Tmax
0.0 0.2 0.4
Variable Importance of Gasoline GAM model at Bronx
Tmax
VOC(Mobile)*Tmax
NOx(Other)*Tmax
VOC(Other)*Tmax
NOx(Mobile)*Tmax
AvgWindSpeed
VOC(Mobile)
DayofYear
NH3(Mobile)*NOx(Mobile)
DayofWeek
AvgRH
0.00 0.25 0.50 0.75 1.00
Variable Importance of Gasoline GAM model at Manhattan
VOC(Mobile)
NH3(Mobile)*NOx(Mobile)
VOC(Other)*Tmax
NH3(Mobile)*SO2(Mobile)
Tmax
DayofYear
AvgWindSpeed
DayofWeek
AvgRH
0.00 0.25 0.50 0.75 1.00
Variable Importance of Gasoline GAM model at Queens
VOC(Mobile)*Tmax
NOx(Mobile)*Tmax
NH3(Mobile)*NOx(Mobile)
Tmax
VOC(Mobile)
NH3(Mobile)*SO2(Mobile)
AvgWindSpeed
VOC(Other)*Tmax
DayofYear
DayofWeek
AvgRH
0.00 0.25 0.50 0.75

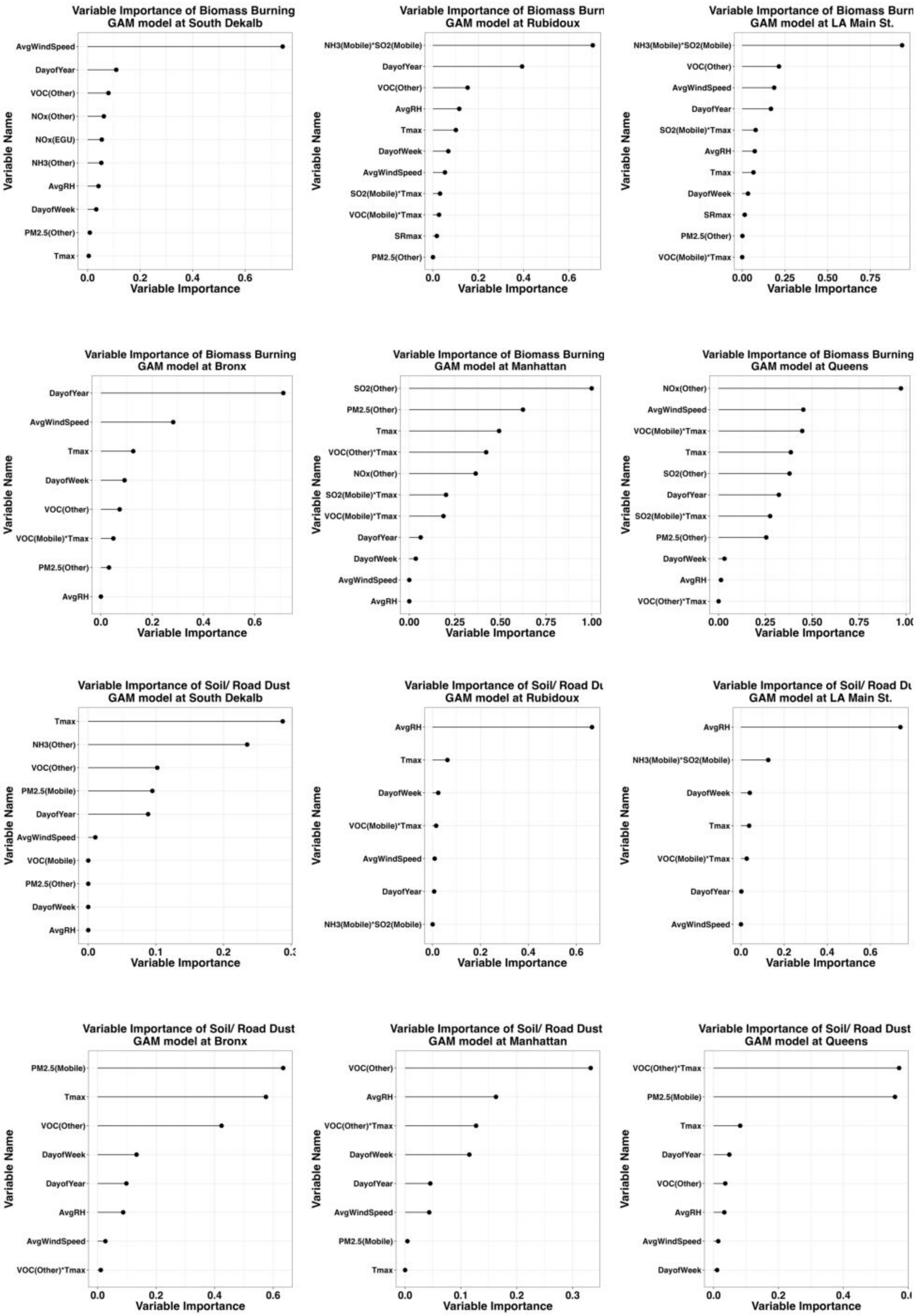

Figure S4. Variable importance of each indicator in source-apportioned $PM_{2.5}$ GAMs at South Dekalb, GA (top, leftmost); Rubidoux (top, middle) and LA Main St. (top, rightmost), CA; Bronx (bottom, leftmost), Manhattan (bottom, middle) and Queens (bottom, rightmost), NY.

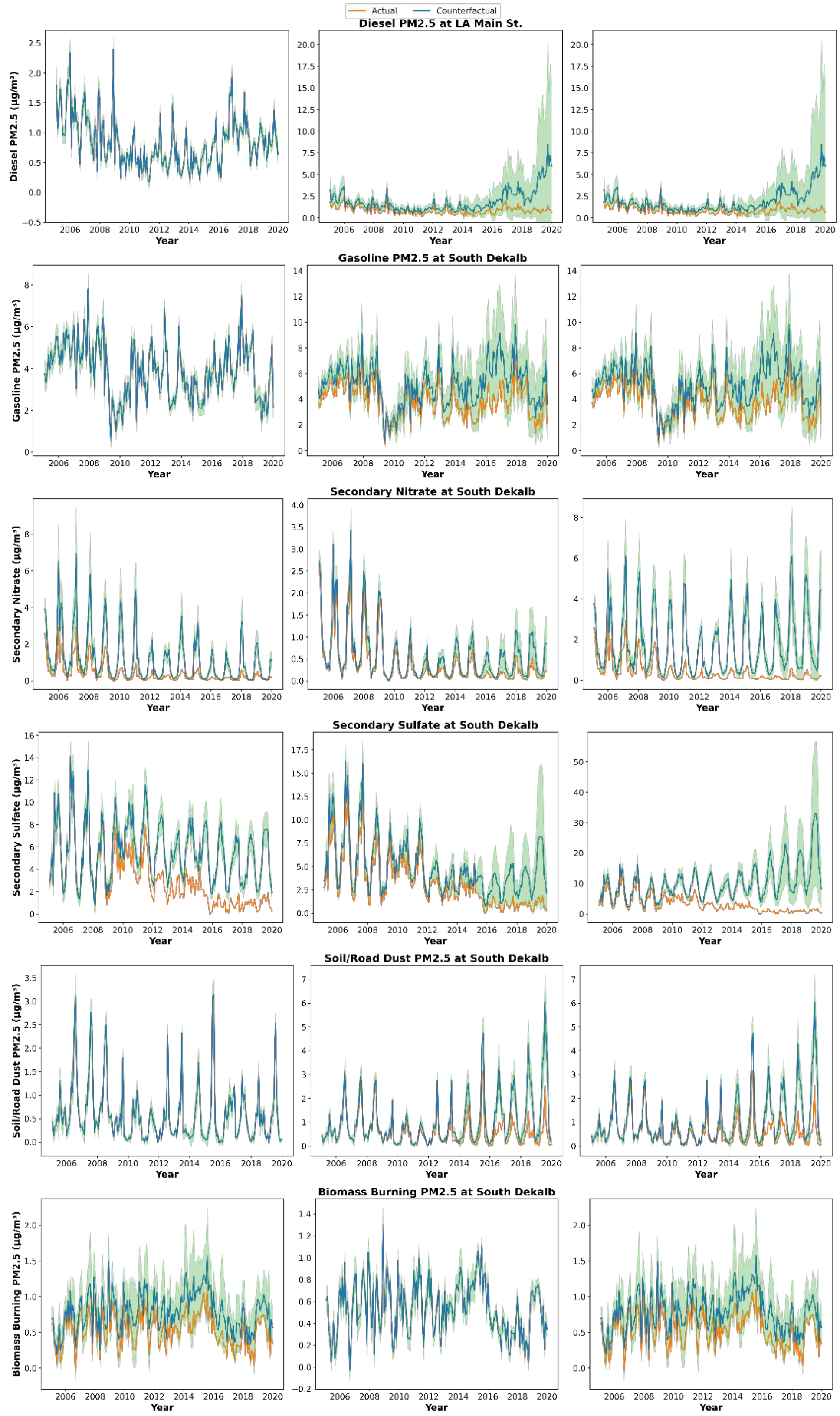

Figure S5. Monthly averaged observations and counterfactual air pollution levels with only counterfactual EGU emissions (left column), only counterfactual mobile emissions (middle

column) and total counterfactual emissions (right column) at South Dekalb, GA. The orange line is observed data, and the blue line is counterfactual air quality data. The green area is the uncertainty.

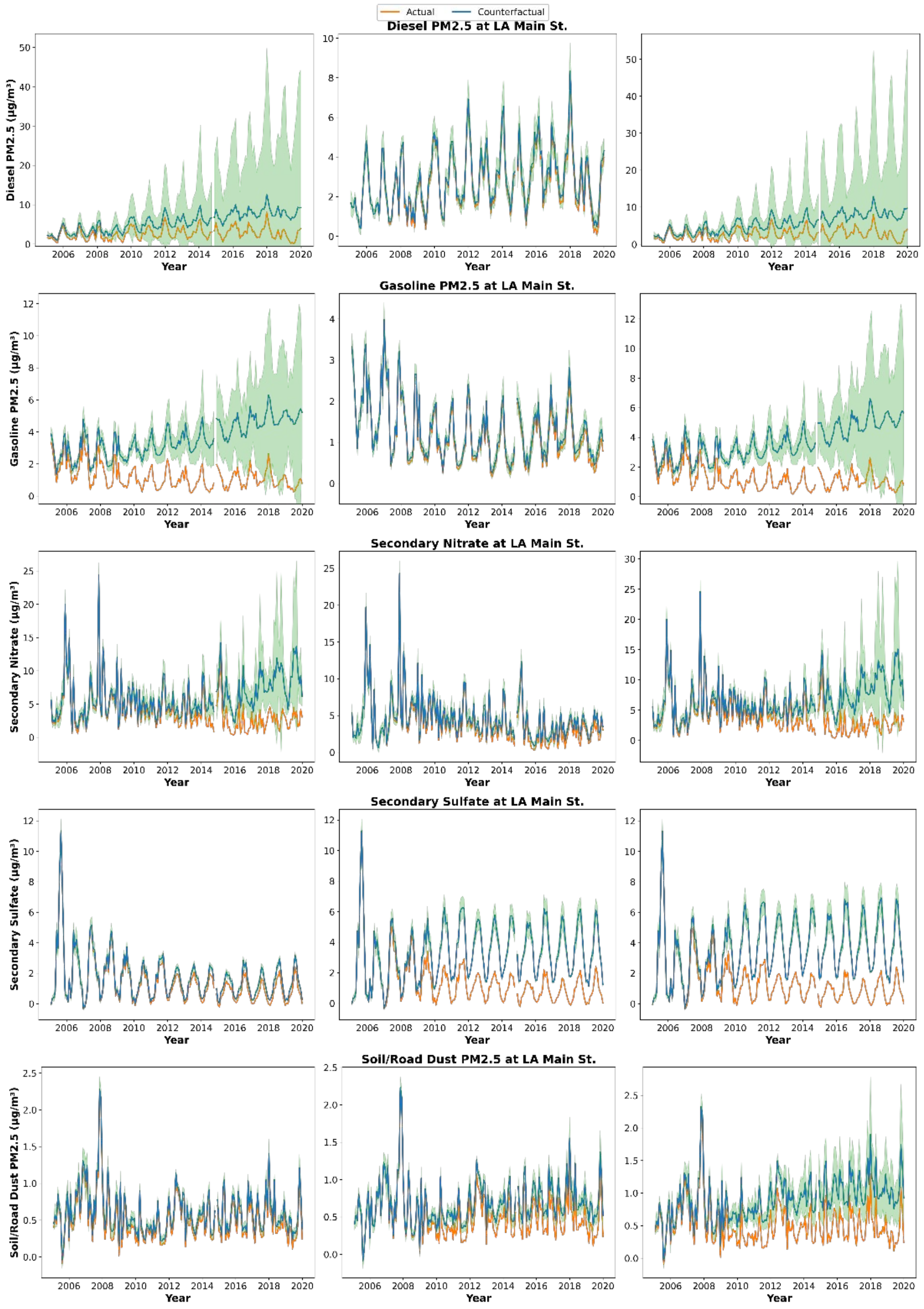

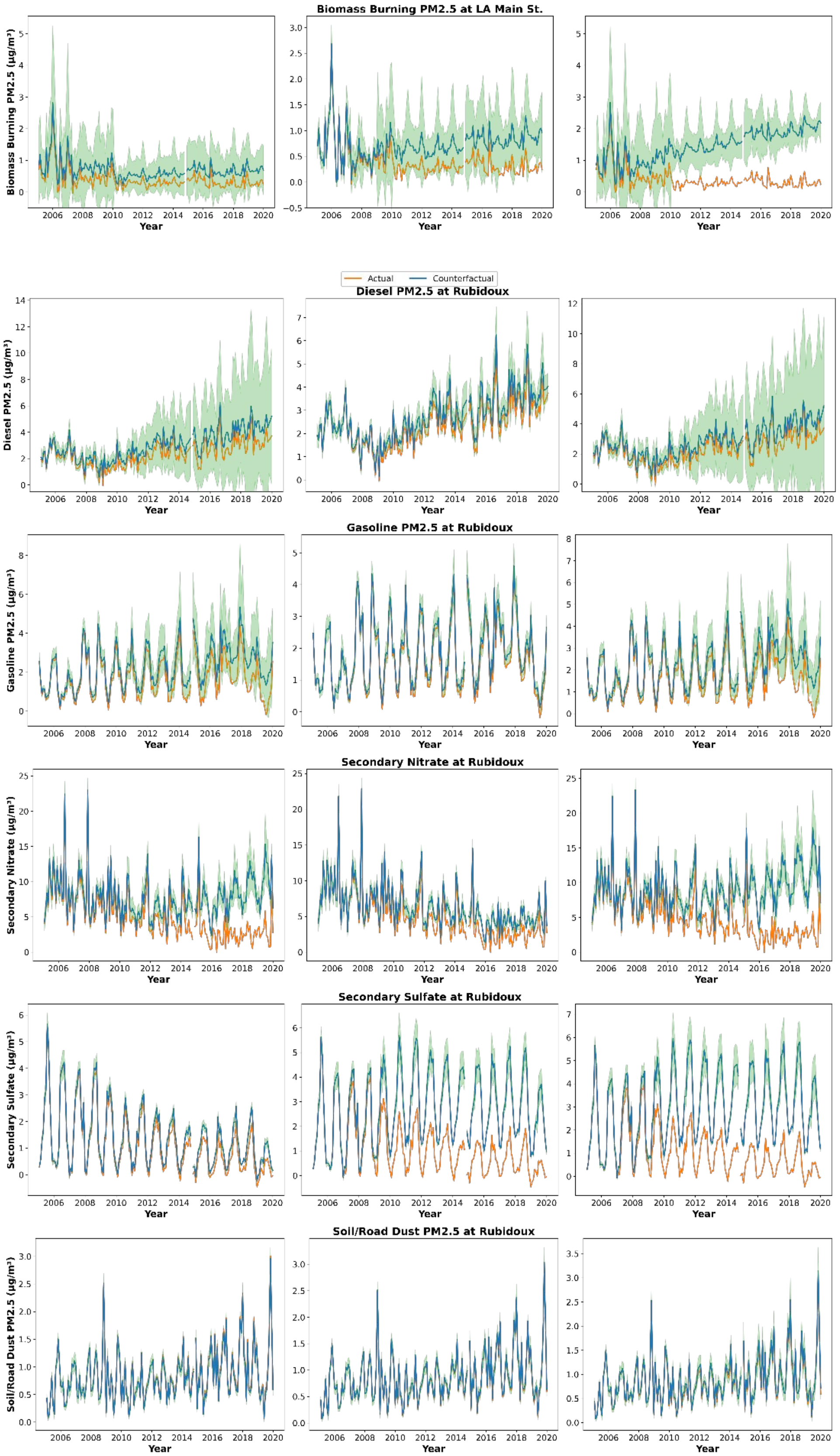
Biomass Burning PM2.5 at LA Main St.
Biomass Burning PM2.5 (μg/m³)
Actual
Counterfactual
Diesel PM2.5 at Rubidoux
Diesel PM2.5 (μg/m³)
Gasoline PM2.5 at Rubidoux
Gasoline PM2.5 (μg/m³)
Secondary Nitrate at Rubidoux
Secondary Nitrate (μg/m³)
Secondary Sulfate at Rubidoux
Secondary Sulfate (μg/m³)
Soil/Road Dust PM2.5 at Rubidoux
Soil/Road Dust PM2.5 (μg/m³)
Year

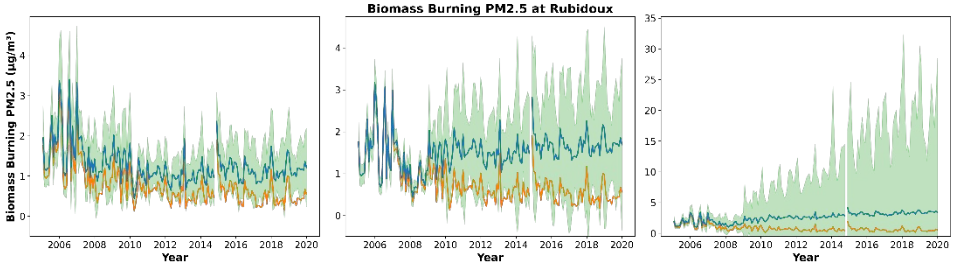

Figure S6. Monthly averaged observations and counterfactual air pollution levels with only counterfactual on-road emissions (left column), only counterfactual port emissions (middle column) and total counterfactual emissions (right column) at LA Main St. (top) and Rubidoux (bottom), CA. The orange line is observed data, and the blue line is counterfactual air quality data. The green area is the uncertainty.

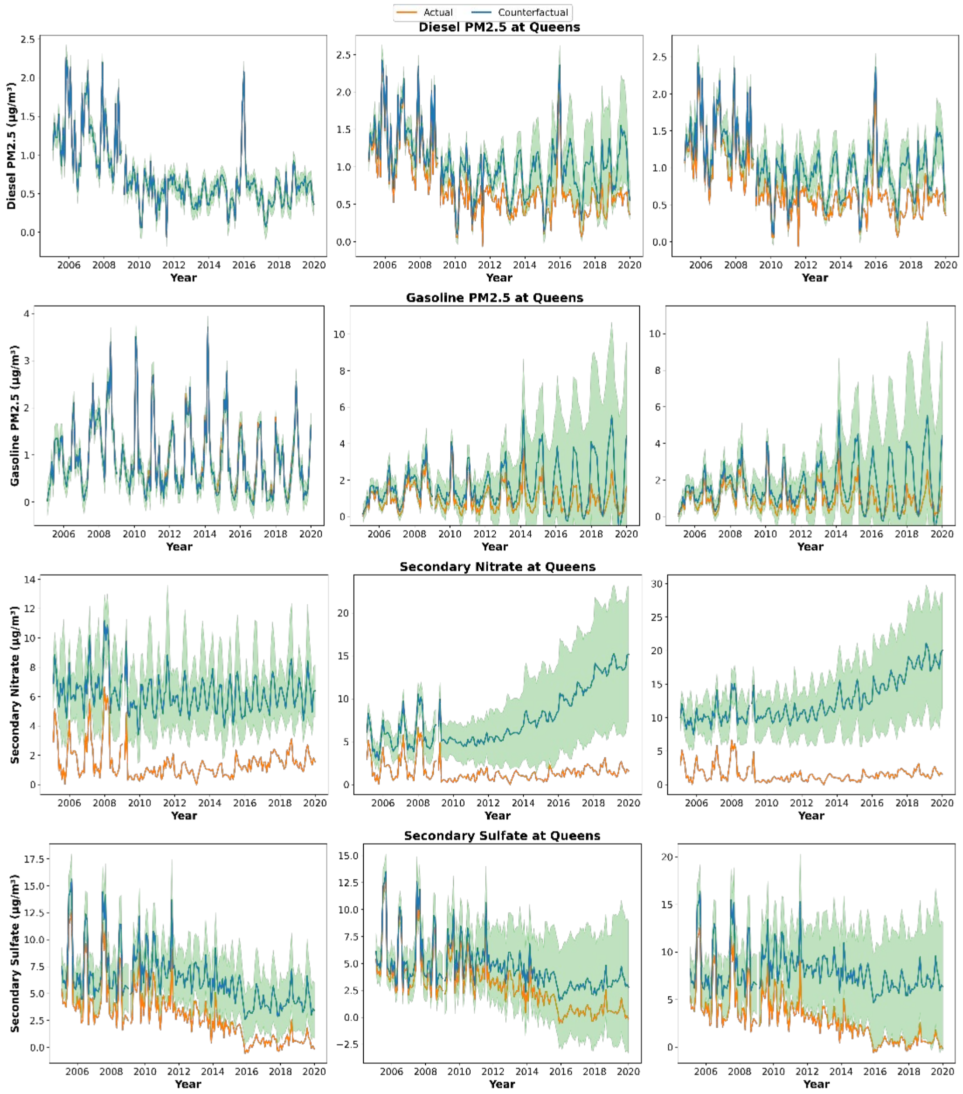

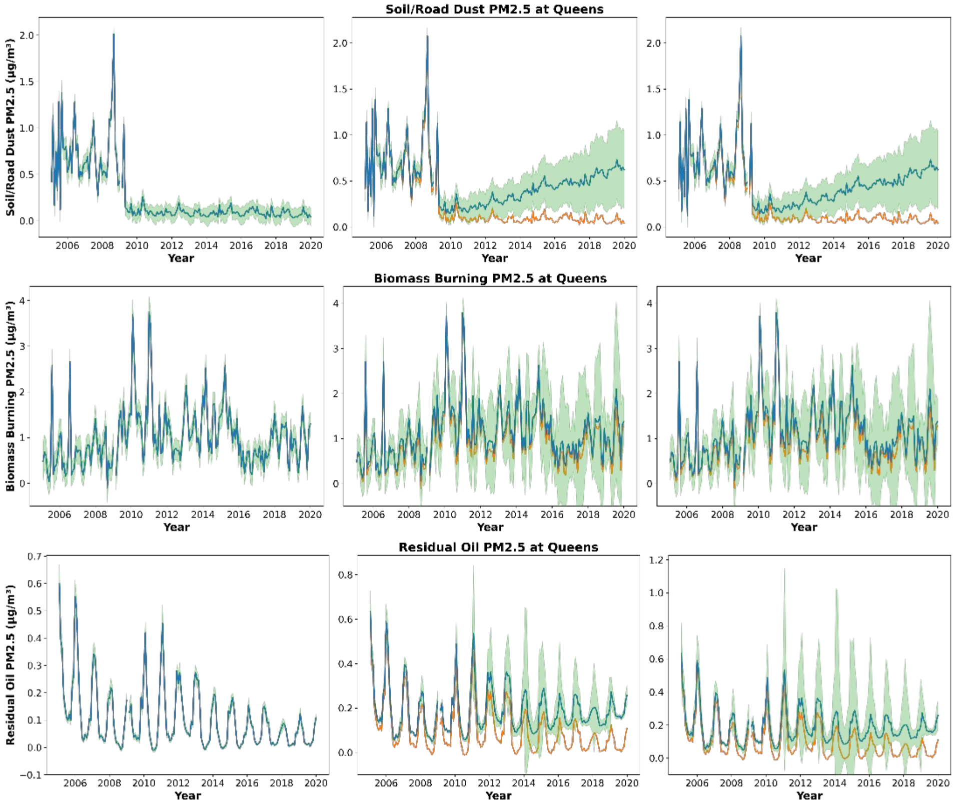
Soil/Road Dust PM2.5 at Queens
Soil/Road Dust PM2.5 (μg/m³)
Biomass Burning PM2.5 at Queens
Biomass Burning PM2.5 (μg/m³)
Residual Oil PM2.5 at Queens
Residual Oil PM2.5 (μg/m³)
Year

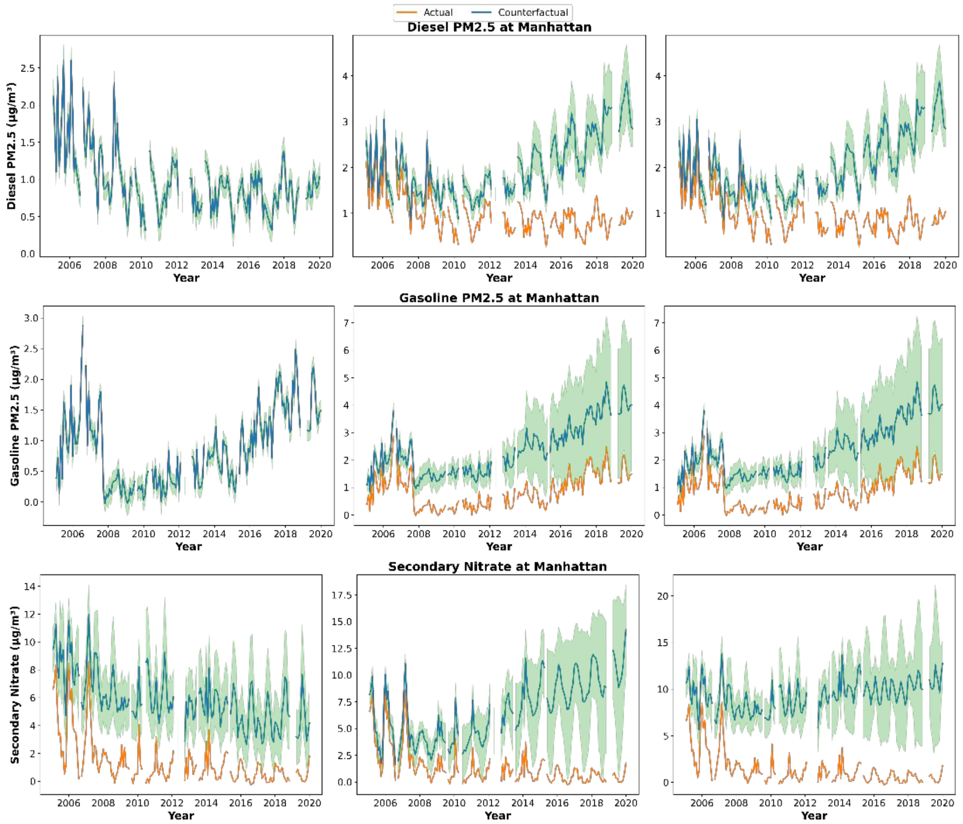
Actual
Counterfactual
Diesel PM2.5 at Manhattan
Diesel PM2.5 (μg/m³)
Gasoline PM2.5 at Manhattan
Gasoline PM2.5 (μg/m³)
Secondary Nitrate at Manhattan
Secondary Nitrate (μg/m³)
Year

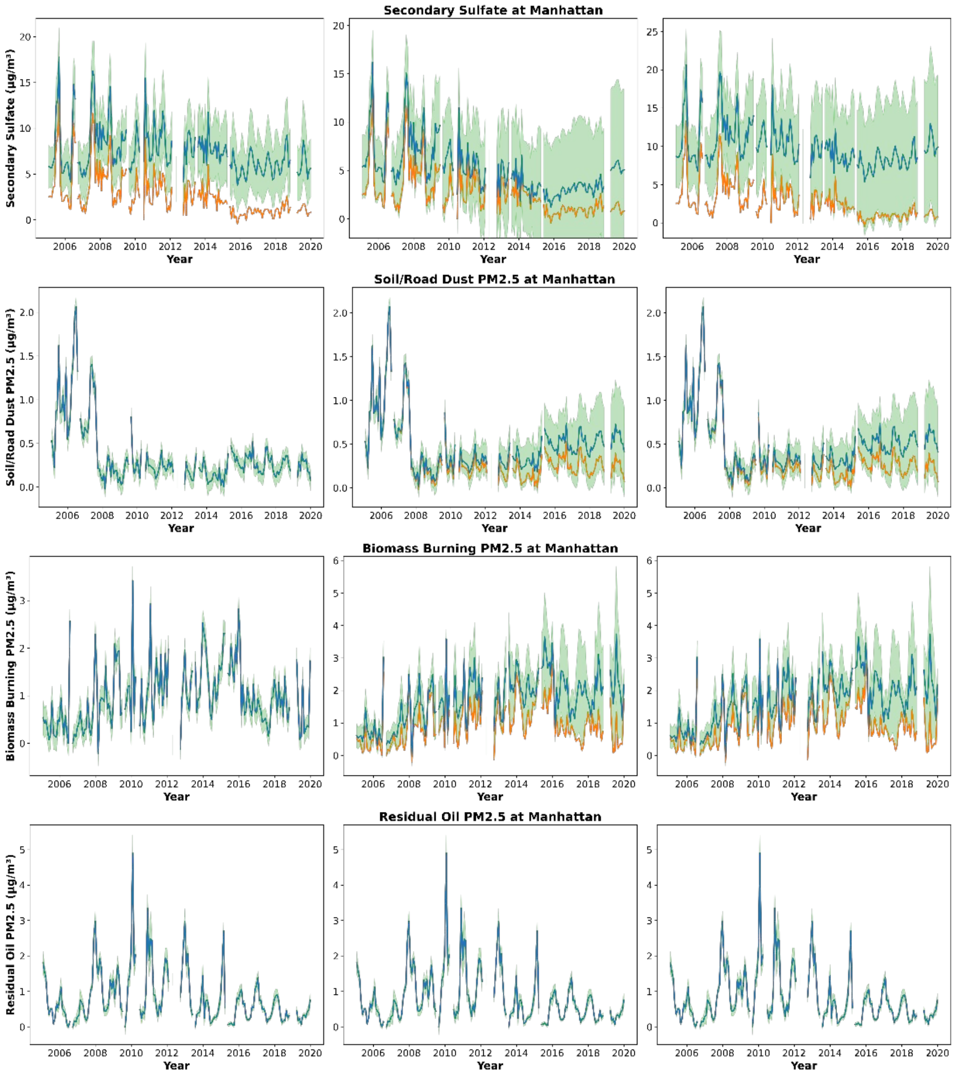
Secondary Sulfate at Manhattan
Secondary Sulfate (μg/m³)
Soil/Road Dust PM2.5 at Manhattan
Soil/Road Dust PM2.5 (μg/m³)
Biomass Burning PM2.5 at Manhattan
Biomass Burning PM2.5 (μg/m³)
Residual Oil PM2.5 at Manhattan
Residual Oil PM2.5 (μg/m³)
Year

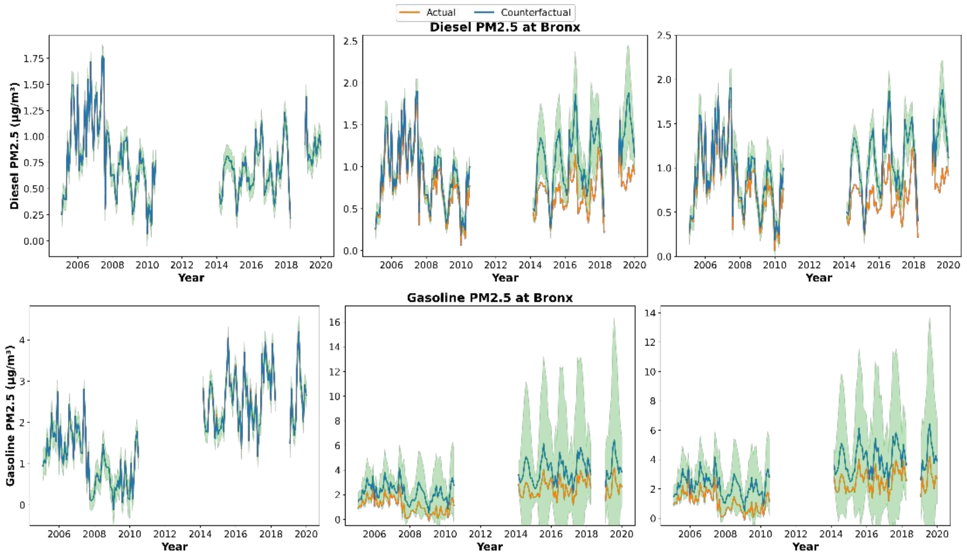
Actual
Counterfactual
Diesel PM2.5 at Bronx
Diesel PM2.5 (μg/m³)
Gasoline PM2.5 at Bronx
Gasoline PM2.5 (μg/m³)
Year

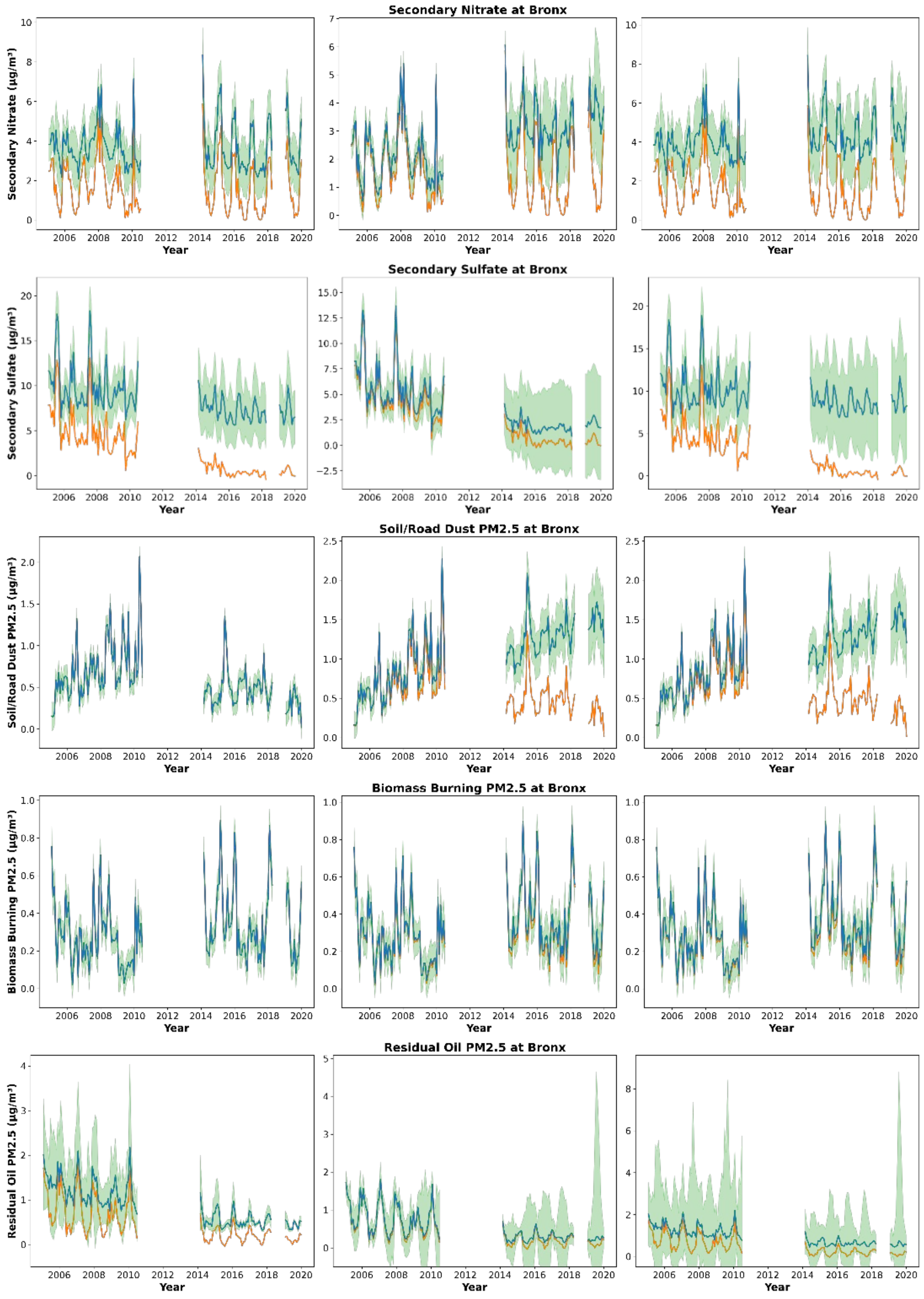

Figure S7. Monthly averaged observations and counterfactual air pollution levels with only counterfactual EGU emissions (left column), only counterfactual mobile emissions (middle column) and total counterfactual emissions (right column) at Queens (top), Manhattan (middle) and Bronx (bottom), NY. The orange line is observed data, and the blue line is counterfactual air quality data. The green area is the uncertainty.

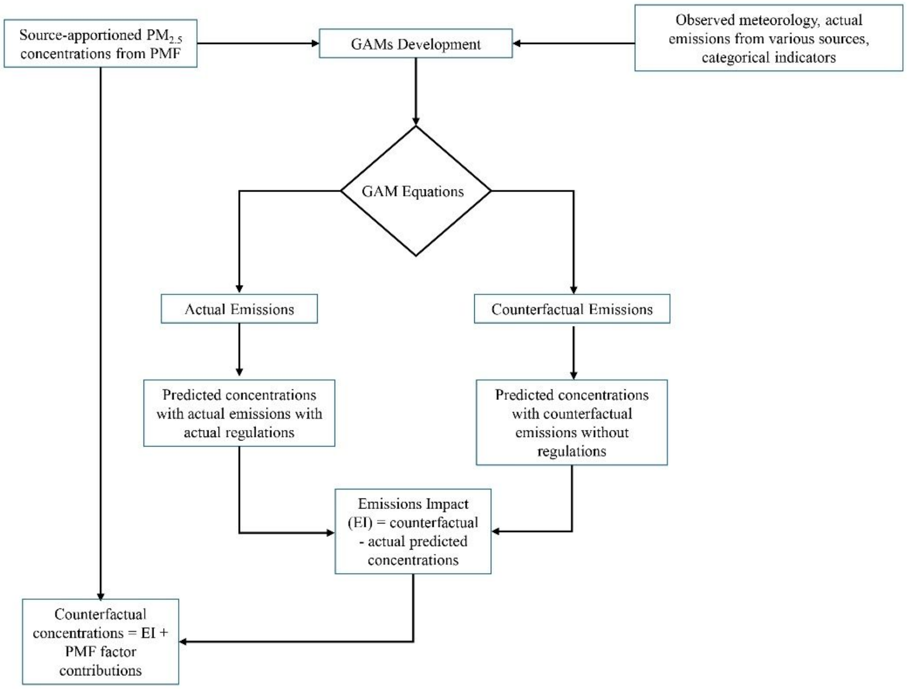

Figure S8. A flowchart of Section 2.2.1.

Table S1. Actual and counterfactual (without all emission controls) concentrations of each PMF factor in Atlanta, New York City, and South Coast Air Basin (SoCAB) in 2019.

Sites/Sources		Diesel	Gasoline	Secondary Nitrate	Secondary Sulfate	Soil/Road Dust	Biomass Burning	Residual oil
**South Dekalb**	Actual	0.85	2.69	0.17	0.99	0.52	0.54	NA
	Counterfactual	5.68	4.17	2.25	20.1	2.14	0.79	NA
**LA N Main St.**	Actual	1.75	0.64	2.72	0.86	0.39	0.26	NA
	Counterfactual	7.93	5.26	9.93	3.81	0.51	2.09	NA
**Rubidoux**	Actual	3.35	0.84	3.13	0.18	0.96	0.43	NA
	Counterfactual	4.43	1.99	11.7	2.60	1.02	3.40	NA
**Bronx**	Actual	0.90	2.59	1.63	0.42	0.29	0.30	0.12
	Counterfactual	1.42	4.23	4.84	8.94	1.48	0.33	0.56
**Manhattan**	Actual	0.90	1.53	0.53	1.03	0.23	0.68	0.35
	Counterfactual	2.28	2.91	14.3	17.6	0.27	0.96	0.36
**Queens**	Actual	0.58	0.91	1.63	0.52	0.06	0.92	0.05
	Counterfactua	1.09	2.11	18.8	7.16	0.64	1.14	0.19

	1							

Table S2. Set of predictors used in each PMF factor's GAM in Atlanta, New York City, and South Coast Air Basin (SoCAB).

**South Dekalb**	Diesel	DOW, DOY, Tmax, WS, RH, SO_2, NOx, and EC emissions from mobile sources, VOC emissions from other sources
	Gasoline	DOW, DOY, Tmax, WS, RH, $PM_{2.5}$, NOx, and VOC emissions from mobile sources
	Secondary Nitrate	DOW, DOY, Tmax, WS, RH, NOx emissions from EGU, the interaction between NOx emissions from mobile sources and Tmax
	Secondary Sulfate	DOW, DOY, Tmax, WS, RH, SO_2 emissions from EGU, the interaction between NH_3 and SO_2 emissions from mobile sources
	Soil/ Road Dust	DOW, DOY, Tmax, WS, RH, $PM_{2.5}$ emissions from mobile sources, NH_3 emissions from other sources
	Biomass Burning	DOW, DOY, Tmax, WS, RH, NH_3, VOC and $PM_{2.5}$ emissions from other sources, NOx emissions from EGU

**SoCAB**	Diesel	DOW, DOY, Tmax, WS, RH, SR, the interaction between SO_2, VOC, NOx, EC emissions from mobile sources and Tmax, the interaction between NOx and NH3 emissions from mobile sources, $PM_{2.5}$ emissions from other sources
	Gasoline	DOW, DOY, Tmax, WS, RH, SR, the interaction between SO_2, VOC, NOx, EC emissions from mobile sources and Tmax, the interaction between NOx and NH_3 emissions from mobile sources
	Secondary Nitrate	DOW, DOY, Tmax, WS, RH, SR, the interaction between NOx, SO_2, EC emissions from mobile sources and Tmax
	Secondary Sulfate	DOW, DOY, Tmax, WS, RH, SR, the interaction between SO_2 emissions from mobile sources and Tmax, SO_2 emissions from other sources
	Soil/ Road Dust	DOW, DOY, Tmax, WS, RH, the interaction between VOC emissions from mobile sources and Tmax, the interaction between SO_2 and NH_3 emissions from mobile sources

	Biomass Burning	DOW, DOY, Tmax, WS, RH, SR, the interaction between VOC emissions from other sources and wind speed, the interaction between SO_2, VOC emissions from mobile sources and Tmax, the interaction between SO_2 and NH_3 emissions from mobile sources, $PM_{2.5}$ emissions from other sources
**New York City**	Diesel	DOW, DOY, Tmax, WS, RH, the interaction between SO_2, NOx emissions from mobile sources and Tmax, $PM_{2.5}$ emissions from other sources.<br>Interaction between SO_2 and NH_3 emissions from mobile sources (only Manhattan)
	Gasoline	DOW, DOY, Tmax, WS, RH, VOC emissions from mobile sources, the interaction between NOx and NH_3 emissions from mobile sources, the interaction between VOC emissions from other sources and Tmax.<br><br>Interaction between NOx, VOC emissions from mobile sources and Tmax (except Manhattan)<br>Interaction between SO_2 and NH_3 emissions from mobile sources (except Bronx)<br>Interaction between VOC emissions from other sources and Tmax (only Bronx)
	Secondary Nitrate	DOW, DOY, Tmax, WS, RH, the interaction between NOx emissions from mobile sources and Tmax.<br><br>NOx emissions from mobile sources, interaction between NOx emissions from EGU sources and Tmax, SO_2 emissions from EGU sources (except Bronx)<br>NOx emissions from EGU sources, NOx emissions from other sources, and interaction between SO_2 emissions from mobile sources and Tmax. (except Manhattan)<br>Interaction between NOx emissions from other sources and Tmax (only Manhattan)<br>Interaction between NOx/ SO_2 and NH_3 emissions from mobile sources (only Queens)
	Secondary Sulfate	DOW, DOY, Tmax, WS, RH, SO_2 emissions from EGU, mobile, and other sources.<br><br>Interaction between SO_2 and NH_3 emissions from mobile sources (only Manhattan)
	Soil/ Road Dust	DOW, DOY, Tmax, WS, RH, VOC emissions from other sources, $PM_{2.5}$ emissions from mobile sources, the interaction between VOC emissions from other sources and Tmax

	Biomass Burning	DOW, DOY, Tmax, WS, RH, $PM_{2.5}$ emissions from other sources, the interaction between VOC emissions from mobile sources and Tmax.<br><br>Interaction between VOC emissions from other sources and Tmax, the interaction between SO_2 emissions from mobile sources and Tmax, SO_2, NOx emissions from other sources (except Bronx)<br>VOC emissions from other sources (only Bronx)
	Residual Oil	DOW, DOY, Tmax, WS, RH, $PM_{2.5}$ emissions from other sources.<br>**MAN**: Interaction between VOC, NOx emissions from other sources and Tmax, SO_2 emissions from other sources, and the interaction between NH_3 and SO_2 emissions from other sources.<br>**QUE**: VOC emissions from other sources, SO_2 emissions from mobile sources, and the interaction between NH_3 and SO_2 emissions from other sources.<br>**BRO**: Interaction between VOC, NOx emissions from other sources and Tmax, NOx and SO_2 emissions from EGU, SO_2 emissions from other sources, and the interaction between SO_2 emissions from mobile sources and Tmax.

DOW: day of week; DOY: day of year; Tmax: Maximum temperature; WS: wind speed; RH: relative humidity; SR: solar radiation; MAN: Manhattan site; QUE: Queens site; BRO: Bronx site.

References:

[1] Gao, Z.; Mei, E.J.; He, X.; Hopke, P.K.; Ebelt, S.; Rich, D.Q.; Russell, A.G. Multicity accountability and uncertainty assessment of the impacts of regulations on air quality in Atlanta, New York City, and Southern California. *Atmos. Environ.* **2025**, 342, 120947; DOI 10.1016/j.atmosenv.2024.120947
